# EPR spectroscopic characterisation of native Cu^II^-binding sites in human serum albumin†

**DOI:** 10.1039/d4dt00892h

**Published:** 2024-07-23

**Authors:** Katrin Ackermann, Dongmei Wu, Alan J. Stewart, Bela E. Bode

**Affiliations:** a EaStCHEM School of Chemistry, Biomedical Sciences Research Complex, and Centre of Magnetic Resonance, University of St Andrews St Andrews KY16 9ST UK; b School of Medicine, Biomedical Sciences Research Complex, and Centre of Magnetic Resonance, University of St Andrews St Andrews KY16 9TF UK beb2@st-andrews.ac.uk ajs21@st-andrews.ac.uk

## Abstract

Human serum albumin (HSA) is the most abundant plasma protein, which functions to transport a large range of ligands within the circulation. These interactions have important implications for human health and disease. The primary binding site for Cu^II^ ions on HSA is known to be the so-called amino-terminal Cu^II^ and Ni^II^ binding (ATCUN) motif. However, the number and identity of secondary binding sites is currently not understood. In this study, we harnessed a suite of contemporary electron paramagnetic resonance (EPR) spectroscopy methods to investigate recombinantly produced constructs of HSA bearing single-histidine knockouts, with the aim to characterise its endogenous Cu^II^ ion binding sites.

## Abstract 2

Using a suite of electron paramagnetic resonance spectroscopy techniques to investigate single-histidine knockout mutants of human serum albumin, a set of three histidine residues can be determined as primary and secondary copper ion binding sites.

## Graphical abstract

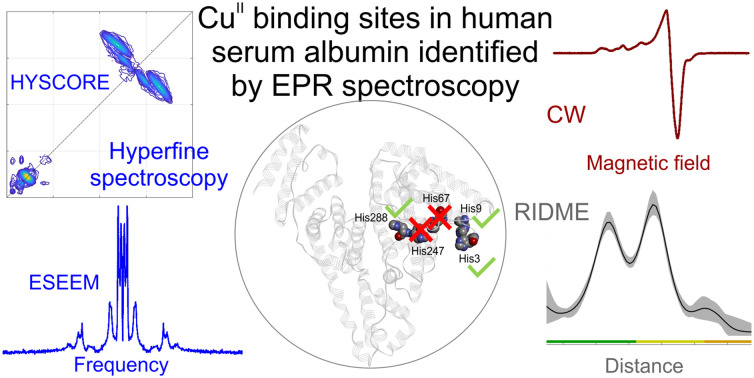

## Introduction

Human serum albumin (HSA) is the most abundant protein in blood plasma with an average concentration of 600–700 μM.^1^ The mature protein has 585 amino acids and a molecular weight of 66.5 kDa, and has been characterised as a monomer with three distinct domains.^1^ HSA has a plethora of ligands, and functional relevance has been attributed to its binding and transport of hormones, drugs, fatty acids and particularly divalent metal ions.^2–4^ For some of these metal ions (*e.g.*, Cu^II^, Zn^II^, Mg^II^, Mn^II^, and Ca^II^), HSA is involved in their transport to cells and tissues and aids in the regulation of cellular metal ion homeostasis,^1,3,5^ while for other metal ions (*e.g.*, Ni^II^, Cd^II^, Co^II^) the interest in their binding to HSA is mainly of toxicological relevance.^2,3^ Increased fatty acid binding to HSA can have important implications in several human diseases,^1,6^ and can be intertwined with concomitant alterations of metal ion binding.^1,5,7^

Free Cu^II^ ions have the potential to generate unwanted reactive oxygen species (ROS) and are therefore meticulously controlled, with HSA binding ∼10–15% of free Cu^II^ in the extracellular environment, while the rest is mainly bound by ceruloplasmin.^8–10^ The interaction of Cu^II^ with serum albumin has been studied for several decades,^11^ and led to the first characterisation of the ATCUN motif, an amino-terminal Cu^II^- and Ni^II^-binding structural feature (also called NTS for N-terminal binding site) involving a critical histidine residue in position three (H3), amongst other characteristics.^9^ The NTS has been identified as the site of highest (picomolar) affinity for Cu^II^ in HSA,^12^ and a study of the isolated N-terminal four residues of HSA (DAHK) in complex with Cu^II^ confirmed its equatorial coordination sphere involving surrounding nitrogen atoms in square-planar orientation and an apically bound water molecule, as well as the silencing effect of the coordination on the redox activity of Cu^II^.^13^ However, due to the high structural flexibility of the native NTS in HSA, the ATCUN residues are mostly missing from high-resolution experimental structures, and a structure directly demonstrating Cu^II^ binding to the HSA ATCUN motif is not available.

Furthermore, the identity of secondary Cu^II^-binding site(s) of HSA remains a matter of active research, with currently no Cu^II^-HSA crystal structures available. Several high-resolution crystal structures of serum albumin with metal ions are available, including Ca^II^,^14^ Zn^II^,^7^ and Co^II^.^1^ One candidate for a secondary Cu^II^-binding site is the multi-metal binding site in HSA, previously introduced as Cd^II^-binding site A,^15^ which was initially suggested to involve histidine residue H247 of HSA domain II and histidine residues H105 and H146 of domain I.^15,16^ Later mutagenesis studies demonstrated the involvement of H67 in addition to H247.^3^ However, relative affinities of this site with respect to Cd^II^ and Cu^II^ are being debated.^15,16^ For Co^II^, three binding sites have been proposed in HSA, including the ATCUN motif, site A, and the as yet unlocalised site B introduced in the Cd^II^ study,^15^ later proposed to involve H9 in domain I,^1^ yet the relevance of site B for Cu^II^-binding is currently not clear. Histidine residue H288 has previously been implicated in binding of Co^II^ and Pt^II^ in HSA,^1,17^ and in binding of Zn^II^ in horse serum albumin (ESA),^18^ and thus serves as another candidate for a secondary Cu^II^ binding site.

Electron paramagnetic resonance (EPR) spectroscopy is exclusively and exquisitely sensitive to paramagnetic centres, such as stable nitroxide radicals introduced at particular sites in biomolecules using site-directed spin labelling,^19^ or paramagnetic metal ions.^20–22^ These can be bound to artificial metal ion binding sites such as the double-histidine motif in proteins,^23^ or to endogenous sites, potentially substituting the native diamagnetic ligand (*e.g.*, Zn^II^), with a paramagnetic one (*e.g.*, Cu^II^).^24^ Due to this sensitivity, EPR is not limited by the size, shape, or complexity of the system under study, nor does it require crystallisation or isotope labelling, which makes it an attractive method for structural biology.

A suite of EPR techniques is available to probe local structural information around paramagnetic centres in metalloproteins, and to measure distances on the nanometre scale between those centres.^24^ The identity of the paramagnetic centre and the binding geometry around it can be characterised by continuous wave (CW) EPR.^25^ Hyperfine spectroscopy, such as electron spin echo envelope modulation (ESEEM)^26–28^ or the corresponding two-dimensional hyperfine sublevel correlation (HYSCORE)^29^ experiment provide information on weakly coupled magnetic nuclei, such as remote nitrogen atoms often attributed to histidine residues involved in metal ion coordination.^28,30–33^ CW EPR has been employed previously to characterise the ATCUN motif in HSA,^34^ and HYSCORE was used to study the NTS peptide of HSA (DAHK).^13^ To complement these hyperfine techniques, pulse dipolar EPR spectroscopy (PDS) experiments, such as the RIDME^35^ method (relaxation-induced dipolar modulation enhancement), are available to determine distances between paramagnetic centres on the nanometre scale.^36,37^

In this study, we aim to identify and characterise the primary and secondary Cu^II^ binding sites of HSA using a holistic approach based on a suite of EPR techniques in combination with recombinantly produced HSA constructs bearing single histidine-knockouts. No high-resolution experimental structure of the full-length protein complexed with Cu^II^ has been reported to date. This strategy can be employed to elucidate the role of individual histidine residues in Cu^II^ binding and obtain insights into the Cu^II^ coordination at individual sites. A better understanding of the occupation of these binding sites will provide a deeper insight into Cu^II^ homeostasis and allow identifying variations observed with fatty acid loading, with potential implications in disease.

## Experimental

### Cloning and site-directed mutagenesis

A synthetic gene containing the coding sequence of HSA (corresponding to residues 25–609 of the albumin preprotein sequence; NP_000468.1, Eurofins Genomics) was amplified and cloned into pPICZαB vector (EasySelect™ Pichia Expression Kit, Invitrogen) for expression in *Pichia pastoris* (Fig. S1†). The gene was inserted in-frame with the N-terminal α-factor secretion signal sequence. Mutant albumin constructs (H3A, H9A, H67A, H247A, H288A) were generated using the GeneArt™ site-directed mutagenesis system (Thermo Fisher Scientific) and Q5 High-Fidelity DNA polymerase (NEB) according to manufacturer protocols. Primers are given in Table S1.† Obtained mutated plasmids were transformed into DH5α™-T1^R^ competent *E. coli* cells and incubated at 37 °C overnight on low salt Luria–Bertani plates containing 25 μg mL^−1^ Zeocin (InvivoGen). Successful mutagenesis was confirmed by DNA sequencing using the α-factor, the 5′ AOX1 and the 3′ AOX1 primers (Table S1†).

### Transformation of DNA into *Pichia pastoris*

Genomic DNA from resultant clones was extracted, linearised using Pme1 (NEB), and transformed into X-33 *Pichia pastoris* competent cells (Thermo Fisher Scientific) by electroporation. Briefly, 6.25 μg of linearised plasmids containing the respective inserts were mixed with 100 μL of X-33 cells and incubated on ice for 5 minutes. The mixtures were then transferred into pre-cooled 0.2 cm electroporation cuvettes. Cells were pulsed (voltage/time constant: 1500 V/5 ms), followed by addition of 1 mL of ice-cold 1 M sorbitol and incubated at 30 °C for 2 hours without shaking. 1 mL of YPD medium was added to the cell cultures and incubated at 30 °C for a further 3 hours without shaking. Cells were then spread on YPDS plates containing 100 μg mL^−1^ Zeocin and incubated at 30 °C for 2 days. To confirm successful integration into the *Pichia pastoris* genome, DNA was extracted (Yeast DNA extraction kit, Thermo Fisher Scientific) and assessed by PCR using the 5′ AOX1 and the 3′ AOX1 primers flanking the insertion site, followed by agarose gel electrophoresis.

### Protein expression and purification

Recombinant wild-type (WT) HSA and mutant forms generated by site-directed mutagenesis were expressed in *Pichia pastoris* and purified as described previously.^1^ Briefly, X-33 cells were grown overnight in 15–50 mL of sterile BMGY medium (buffered minimal glycerol medium containing histidine) containing 100 mg mL^−1^ zeocin at 28 °C with shaking at 200 rpm. This starter culture was used to inoculate 350–500 mL of sterile BMGY medium and grown overnight until the culture reached log phase growth (OD_600_ = 2–6). Cells were harvested and resuspended into 2–4 L of medium. This culture was grown for 5 days, and protein expression was induced by adding 0.5% methanol every 24 h.

The supernatant containing the secreted overexpressed protein was obtained by centrifugation and concentrated before purification using a HiTrap Blue HP column (Cytiva) followed by size exclusion chromatography on a HiLoad Superdex-75 column (Cytiva). Final purity of the protein was assessed to be >95% by SDS-PAGE. Protein intact mass was confirmed by our in-house mass spectrometry service.

### EPR sample preparation

WT (plasma-purified) HSA (Sigma-Aldrich; cat. no. A1887), recombinantly-produced WT HSA (rHSA) as well as mutant HSA variants were dialysed against Milli-Q water and lyophilised. Stock solutions of 1 mM HSA were prepared by resuspension in protonated or deuterated buffer A (50 mM Tris (Merck), 50 mM NaCl (Fisher Scientific), pH 7.4). Albumin concentration was determined by measuring the absorbance at 280 nm based on an extinction coefficient of 34 445 M^−1^ cm^−1^. Stock solutions of CuCl_2_ (Thermo Scientific Chemicals) at 1 mM or 5 mM concentration were prepared freshly in buffer A for protonated samples, or in D_2_O (Merck) for deuterated samples. Protonated and deuterated samples were prepared with a final volume of 20 and 70 μL, respectively, at 250 μM final protein concentration, with varying concentrations of CuCl_2_ (0 to 5 molar equivalents), and 50% (v/v) cryoprotectant (protonated (Alfa Aesar) or deuterated (Cortecnet) glycerol). Samples were immediately snap-frozen in liquid nitrogen. A detailed sample list can be found in Table S2.†

### CW EPR

CW EPR spectra of deuterated samples were obtained at 120 K with a Bruker EMX 10/12 spectrometer running Xenon software and equipped with an ELEXSYS Super Hi-Q resonator at an operating frequency of ∼9.50 GHz with 100 kHz modulation. Temperature was controlled with an ER4141 VTM Nitrogen VT unit (Bruker) operated with liquid nitrogen. CW spectra of the pseudo-titration samples (0.5–5.0 molar equivalents of Cu^II^ added) were recorded using a 1600 G field sweep centred at 3100 G, a time constant of 20.48 ms, a conversion time of 20.62 ms, and 2667 points resolution. CW spectra of the recombinantly produced HSA samples with 2 molar equivalents of Cu^II^ were recorded using a 1800 G field sweep centred at 3100 G, a time constant of 20.48 ms, a conversion time of 20.62 ms, and 3000 points resolution. An attenuation of 10.0 dB (20 mW power) and a modulation amplitude of 3 G were used for all recordings. All CW spectra were phase- and background-corrected and the double integral was obtained using the Xenon software. Simulations and fitting of field-corrected CW EPR spectra (DPPH standard) were performed using the pepper and esfit functions in EasySpin.^38^

### Pulse EPR experiments

Pulse experiments were performed at X- (9–10 GHz) and at Q-band (34 GHz) frequencies, both operating on a Bruker ELEXSYS E580 spectrometer, with probe-heads supporting a split ring resonator (4118X-MS3) for X-band and a 3 mm cylindrical resonator (ER 5106QT-2w) for Q-band, respectively. Pulses were amplified by pulse travelling wave tube (TWT) amplifiers (Applied Systems Engineering) with nominal output of 1 kW and 150 W at X- and Q-band, respectively. Temperature was controlled *via* cryogen free variable temperature cryostats (Cryogenic Ltd) operating in the 3.5–300 K temperature range.

### Hyperfine spectroscopy (protonated samples)

3-Pulse ESEEM^26–28^ spectroscopy was performed at 30 K at X-band frequencies (∼9–10 GHz) and on the maximum of the field-swept spectrum with a pulse length of 16 ns for π/2 pulses and the inter-pulse delay *τ* set at the blind spot of the proton (∼220 ns corresponding to ∼3 times the inverse of the proton Larmor frequency, with *τ*_1(H)_ ∼73 ns). The delay *T*, set at 280 ns, was incremented with a dwell time Δ*T* of 8 ns and a 4-step phase cycle was used. Four *τ* values were recorded, whereby the inter-pulse delay was incremented by 36 ns (∼0.5 × *τ*_1(H)_); the third *τ* was selected for further processing of the ESEEM spectra and for setting up the HYSCORE experiment (see below). Data were analysed by fitting an exponential decay background function to the (phase-corrected) raw data, subtracting the raw data by this background function, and then dividing the difference by the background function, thus retaining amplitude information after fast Fourier transformation (FFT) similar as described previously.^39^ The resulting trace was further subjected to a Hamming window, zero-filling and FFT, before obtaining the absolute (or magnitude) spectrum.

HYSCORE^33^ spectroscopy was performed at 15 K at X-band frequencies (∼9–10 GHz) and on the maximum of the field-swept spectrum with a pulse length of 16 and 32 ns for π/2 and π, respectively, *τ* set at the blind spot of the proton (∼290 ns) as chosen from the 3-pulse ESEEM, *t*_1_ = *t*_2_ = 56 ns, and a 4-step phase cycle. Data were processed and analysed using HYSCOREAN,^30^ employing Hamming apodization, zero-filling, 3^rd^ order polynomial background correction, and diagonal and anti-diagonal spectral symmetrisation, keeping a similar amount of noise for each spectrum by adjusting the minimum contour level percentage accordingly.

### Relaxation time measurements (deuterated samples)

Relaxation time measurements were recorded at 30 K at Q-band frequencies (34 GHz) and at the field position corresponding to the maximum of the Cu^II^ field-swept spectrum. Transverse dephasing times *T*_2_ (or *T*_m_) were determined from 2-pulse electron–spin echo decay experiments recorded with a *τ* of 200 ns, using 16 and 32 ns π/2 and π pulses, under the stretched exponential approximation. Longitudinal relaxation times *T*_1_ were estimated from 3-pulse inversion recovery measurements recorded with a *τ* of 800 ns, an 8 ns inversion pulse and 16 and 32 ns observer pulses (π/2 and π, respectively), under the biexponential approximation.

### RIDME measurements (deuterated samples)

The 5-Pulse RIDME experiments^36^ were recorded at 30 K at Q-band frequencies (34 GHz), using the pulse sequence (π/2–*τ*_1_–π–(*τ*_1_ + *t*)–π/2–*T*_mix_–π/2–(*τ*_2_–*t*)–π–*τ*_2_–echo) at the field position corresponding to the maximum of the Cu^II^ field-swept spectrum with 8-step phase cycling, a *τ*_1_ of 400 ns, a *τ*_2_ of 2500 ns, a shot repetition time (SRT) of 500 μs, and a critically coupled resonator (high Q). Measurements were recorded with mixing times corresponding to 0.7 × *T*_1_ (longitudinal relaxation time as estimated under the biexponential approximation). RIDME data were analysed using the ComparativeDEERAnalyzer^40,41^ (CDA) 2.0 within DeerAnalysis2022.^42^ Full CDA reports are provided in the ESI and the underpinning data.†

## Results & discussion

In this study, we first investigated Cu^II^ binding to HSA using CW EPR. Together with hyperfine techniques, these EPR methods can also provide evidence on the chemical environment of the bound Cu^II^, allowing some characterisation of the binding sites.

To this end, a pseudo-titration series of plasma-purified HSA with varying amounts of Cu^II^ was generated, where each titration point is a distinct sample.^24^

**Fig. 1:**
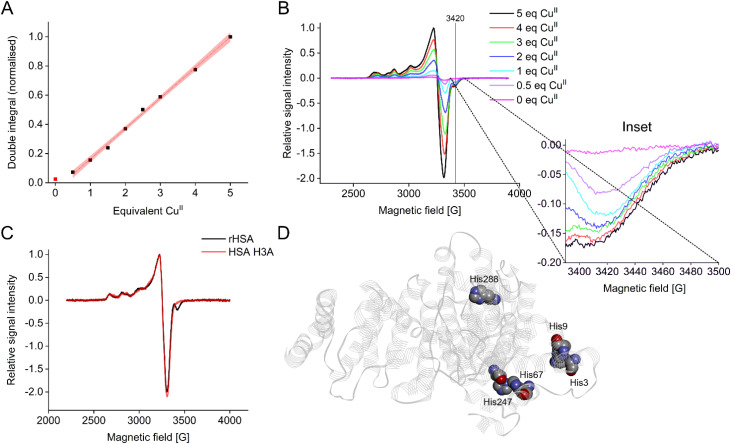
(A) Double integration of Cu^II^ spectra for the HSA pseudo-titration series, normalised. (B) Selected individual CW EPR spectra, normalised and overlaid. Note the distinct, almost constant spectral component at ∼3420 G (see Inset) corresponding to a lower *g* value for the first (high affinity) binding site. (C) Overlay of normalised CW EPR spectra for recombinant WT HSA (rHSA) and the recombinantly produced HSA variant H3A, each with 2 molar equivalents of Cu^II^. The H3A variant does not show the spectral component for the high affinity binding site observed in (B), strongly indicating that H3 is involved in Cu^II^ coordination of this site. (D) Structure of HSA showing locations of studied histidine residues. The cartoon was drawn from structural coordinates obtained from the RSCB Protein Data Bank (PDB ID: 5IJF^18^). Note that the sidechains of His3 and His9 were partially missing from the X-ray structure, and were modelled using Discovery Studio Visualizer v24.1.0.23298 (Dassault Systèmes Biova Corp.). For more details see Fig. S10.†

Double integration of CW EPR spectra showed a linear increase of the Cu^II^ signal from 0.5 to the maximum of 5.0 molar equivalents of Cu^II^ added, suggesting that all added Cu^II^ was bound (Fig. 1A). There was also a small but measurable Cu^II^-signal without addition of Cu^II^ (red point in Fig. 1A), corresponding to ∼1/3 to 1/4 binding site (as estimated from the double integral), which suggested that some Cu^II^ had been co-purified with HSA from plasma.

Individual CW EPR spectra (Fig. 1B) revealed the presence of a distinct spectral component at approximately 3420 G already from the addition of 0.5 molar equivalents of Cu^II^. Thus, this initial signal must be directly associated with the binding site of highest affinity, presumably at the N-terminus (ATCUN motif), exhibiting a lower *g* value than the lower-affinity sites.^34^ Detailed studies of the correlation of CW EPR parameters (in particular *A* and *g* values),^43^ were used to establish the square planar arrangement of the CuN_4_ site present in the ATCUN motif.^34^

Upon addition of more than 1 molar equivalent of Cu^II^ this feature did not grow any further, suggesting that the corresponding site was already fully occupied. Additional variations in spectral line shapes (for individual CW spectra see Fig. S2†) further demonstrated the simultaneous presence of multiple Cu^II^ species, suggesting more than one Cu^II^ coordination mode involving nitrogen (histidine/imidazole; Tris buffer) and oxygen atoms (2N2O; 3N1O).^31,43,44^

Even though the involvement of histidine residues in Cu^II^ binding of HSA is beyond doubt,^2,34^ we did not observe any clear ^14^N superhyperfine (SHF) couplings in the CW EPR spectra as seen previously for another Cu^II^-binding plasma protein.^24^ This might suggest close proximity of Cu^II^-binding sites in HSA leading to dipolar line broadening and thus, loss of resolution of SHF coupling, or the involvement of other complicating factors, such as anisotropic hyperfine interactions. A previous CW EPR study on Cu^II^ binding of albumin and Cu^II^-histidine complexes could demonstrate the enhanced resolution of the superhyperfine splittings using lower frequency measurements (S-band, 3.87 GHz).^34^

To further investigate the identity of the high-affinity site yielding the spectral component around 3420 G in CW EPR, a set of HSA histidine mutants (H3A, H9A, H67A, H247A, and H288A, Fig. 1D) was generated and subjected to cryogenic CW EPR measurements in presence of two molar equivalents of Cu^II^. Results clearly demonstrated that the high-affinity binding site was directly related to the histidine residue at position 3 (H3), as only in the H3A mutant the associated spectral component was absent (Fig. 1C and Fig. S3†), confirming previous data.^9,34,45^ The Cu^II^ coordinated by the ATCUN motif has been shown to be square planar,^34^ with an apical water molecule observed in the DAHK peptide.^13^ Square planar Cu^II^ is expected to show lower *g*_⊥_ values (and thus, CW EPR signals at higher field) than tetrahedral, octahedral and Jahn–Teller distorted geometries. This was confirmed by simulations (Fig. S4 and Table S3†).

Additionally, relaxation time measurements were performed on the samples of the pseudo-titration series to investigate the change in relaxation behaviour between 0.5 and 5.0 molar equivalents of Cu^II^. To ensure relevance for the timings of the RIDME experiment, deuterated samples were used for these measurements. While the general trend was consistent, with continuously faster inversion recovery (*T*_1_ relaxation) and faster two-pulse electron spin echo decay (*T*_2_ relaxation) with increasing amounts of Cu^II^ present, substantially different relaxation times were found between the 0.5 molar equivalents of Cu^II^ and the samples with higher Cu^II^ load, suggesting that the N-terminal high affinity site might be less dynamic even in frozen solution (Fig. S5 and Table S4†). However, the fact that we observe a less dynamic Cu^II^ centre when the square planar ATCUN motif is mainly occupied is coincidental, not causal, *i.e.* the determining factors are currently unclear.

**Fig. 2:**
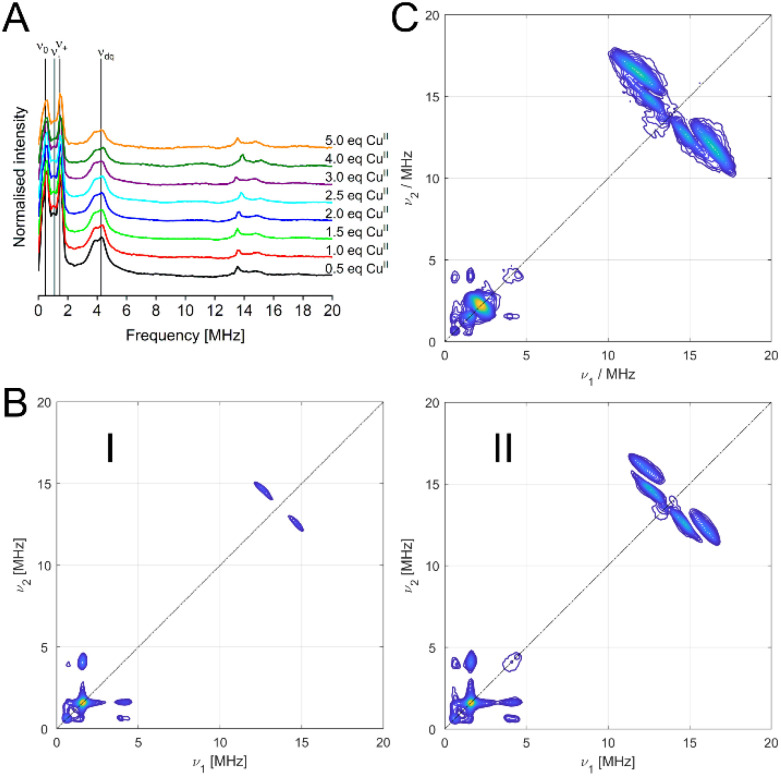
(A) Stacked plots of three-pulse ESEEM data for the Cu^II^ pseudo-titration series, obtained at X-band frequencies (9.5 GHz) and on the maximum of the field-swept spectra. Vertical lines indicate the frequencies expected in the presence of two (or more) histidine residues (NQI: ν_0_, ν_+_, ν_−_; DQ: ν_dq_). (B) Representative (+,+) HYSCORE spectra for HSA with 0.5 (1) and 5.0 (II) molar equivalents of Cu^II^, obtained at X-band frequencies (9.5 GHz) and on the maximum of the field-swept spectra. (C) (+,+) HYSCORE spectrum for HSA H3A with 0.9 molar equivalents of Cu^II^, obtained at X-band frequencies (9.5 GHz) and on the maximum of the field-swept spectrum.

Data from hyperfine spectroscopy provided additional information on the chemical environment of the metal ion binding sites in HSA. Three-pulse ESEEM was used to identify weakly coupled nuclei.^33^ Directly coordinated ligand atoms to the paramagnetic Cu^II^ are commonly too strongly coupled to be observed in ESEEM under our conditions. ESEEM spectra for all samples of the pseudo-titration series revealed very similar frequencies typical for nuclear quadrupole interactions (NQI, narrow lines between ∼0.5 to 2 MHz in Fig. 2A and Fig. S6†) of ^14^N and the double quantum transition (DQ, a broader line at around 4 MHz in Fig. 2A and Fig. S6†) of remote ^14^N in the imidazole ring of histidine residues,^24,33,44^ thereby confirming the involvement of histidine residues in Cu^II^ coordination of HSA.

While a distinct DQ peak was exhibited for all samples of the pseudo-titration with Cu^II^, its amplitude, which is directly correlated to the number of histidines involved in the coordination of the metal ion at a particular site,^32^ was gradually decreasing with increasing Cu^II^ load. This suggested that the average number of histidine residues available per site was also decreasing, or in other words, data indicated that weaker binding sites with fewer histidines were bound at higher Cu^II^ concentration. HSA has a total of 16 histidine residues, and besides the high affinity N-terminal metal ion binding site around H3 a number of additional lower affinity sites have been postulated for various metal ions, some of them including one or more histidine residues.^18^

Three-pulse ESEEM for the recombinant H3A mutant confirmed that the NQI and DQ peaks characteristic for histidine residues in the binding environment were still present even if the N-terminal ATCUN motif is disrupted by removing the respective histidine residue, suggesting that additional histidine residues were involved in Cu^II^ coordination of the lower affinity binding sites (Fig. S7†). However, we cannot exclude that a second histidine residue is involved in Cu^II^ binding at the N-terminal high affinity site which would give rise to a similar ESEEM spectrum. Speculatively, this second histidine residue could be H9, as it is in very close proximity to H3, and a variation of the Co^II^-binding site B, termed site B′, has been shown to involve besides H9 the aspartate residue at position 1 instead of the one at position 13, meaning H9 can interact directly with residues of the NTS.^1^ Therefore, joint participation of H3 and H9 in the NTS at low Cu^II^ load would explain the larger DQ peak observed for the 0.5 molar equivalents of Cu^II^ sample, while at higher Cu^II^ load H9 is involved in a different site (*vide infra*). However, we currently do not have sufficient information to distinguish between this model and other weaker binding sites involving fewer histidine residues.

ESEEM data were further supported by results from the corresponding two-dimensional HYSCORE experiments, with all spectra of the Cu^II^ pseudo-titration series exhibiting highly similar combination peaks for weakly coupled ^14^N (∼0.5–4 MHz, Fig. 2B and Fig. S8†), while the amount of water-coordinated Cu^II^ was found to be increasing with Cu^II^ load (splitting in the signal at ∼14 MHz).

Interestingly, the HYSCORE spectrum of the H3A mutant with 0.9 molar equivalents of Cu^II^ (Fig. 2C and Fig. S9†) showed weaker nitrogen signals compared to the 1 molar equivalent spectrum of the pseudo-titration series, emphasising the role of the H3 for the intensity of the nitrogen peaks. Notably, the H3A also displayed a much stronger signal from water-coordinated Cu^II^ strongly indicating the absence of an apical water molecule in HSA, in contrast to the DAHK peptide.^13^

With the combined results from the CW EPR and hyperfine spectroscopy, we have been able to confirm that the histidine at position 3 in the ATCUN motif is the Cu^II^-binding site of highest affinity in HSA, exhibiting a distinct spectral component with lower *g* value, in line with a previous EPR study.^34^ In addition, it could be shown that other histidine residues are involved in further binding sites. However, the actual identity of the residues involved has not yet been resolved. For Co^II^, for which HSA is the main carrier in the circulation, several other binding sites in HSA have been identified, including site A (involving histidine residues H67 and H247), site B (involving H9), and a secondary site involving H288, which has also been shown to play a role in Zn^II^ binding in ESA.^1,18^

**Fig. 3:**
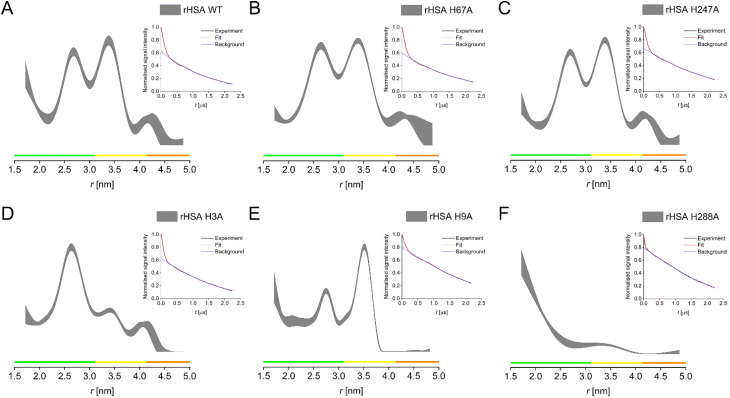
PDS (RIDME) distance distributions for recombinantly produced HSA WT (A), H67A (B), H247A (C), H3A (D), H9A (E), H288A (F). Distance distributions are given as 95% confidence estimate. Colour bars represent reliability ranges (green: shape reliable; yellow: mean and width reliable; orange: mean reliable). Insets show raw RIDME traces (black) with fit (red) and background (blue) for each mutant.

In the following, single His → Ala HSA constructs for these four candidate histidine residues (H9, H67, H247, H288), in addition to the NTS mutant (H3A), were employed to investigate the effect of the removal of individual histidines on the resulting distance distribution in PDS (RIDME) experiments compared to the HSA WT (Fig. 3 and Table S5†). Two main distances were observed for the recombinantly expressed HSA WT, at ∼2.7 nm and ∼3.5 nm (Fig. 3A). Distance distributions obtained for the HSA H67A and the HSA H247A mutants were highly similar (Fig. 3B and C), exhibiting the nearly identical main distances as the HSA WT. This suggested that these two histidine residues did not majorly contribute to the observed distributions and thus, were not significantly involved in high affinity Cu^II^-binding sites, contrarily to what was proposed previously.^15,16^ In contrast, elimination of H3 dramatically altered the resulting distance distribution, with the disappearance of the longer distance, while the ∼2.7 nm distance remained (Fig. 3D), again confirming the involvement of the ATCUN motif in HSA high-affinity Cu^II^-binding. Interestingly, a similar effect was observed for the HSA H9A mutant, in this case with the shorter distance becoming drastically reduced in amplitude, while the longer distance remained untouched (Fig. 3E). This suggested the involvement of H9 in Cu^II^-binding, which may be able to coordinate Cu^II^ in a similar fashion to the proposed site B for Co^II^, which involved an additional O-ligand from the aspartate residue 13.^1^ Another dramatic alteration of the distance distribution was obtained for the H288A mutant, where both, the ∼2.7 and the ∼3.5 nm distances were substantially, if not completely, reduced, with only the indication of a very short distance present remaining (Fig. 3F). Note that all raw RIDME traces are clearly modulated (insets Fig. 3) and were processed with zero-bias.

To rationalise these data, we hypothesised the presence of three high-affinity Cu^II^-binding sites in HSA, involving a triangle of three histidine residues, which must involve three distances in between these residues. RIDME data provided evidence that of the two main distance peaks observed in the WT, the longer distance is associated with the H3 residue, while the shorter one is associated with the H9 residue. The third distance of the proposed triangle is supposedly so short that it is at or beyond the limit of PDS capabilities (lower distance limit of ∼1.5 nm), which would likely be the distance between the H3 and the H9 residues. This was supported by the increase in distance probabilities towards the lower distance limit in all distance distributions. It also further supports our speculation that H9 might be involved in Cu^II^ binding at the ATCUN site as second histidine at low Cu^II^ load, explaining the larger ESEEM DQ peak at low Cu^II^ equivalents, while it becomes a lower-affinity binding site on its own when more Cu^II^ is present.

RIDME data for H288 confirmed this residue as the third vertex of the triangle. H288 was found to be a secondary binding site for Zn^II^ in ESA, together with H157 and E153.^18^ While H157 is not conserved in HSA, which has a phenylalanine in position 157, it can be speculated that the lower coordination number is sufficient for Cu^II^ binding at this site in HSA. Thus, based on RIDME distance distributions, we confirmed a set of three sites involving histidine residues H3, H9, and H288 (Fig. S10†), as the sites of high-affinity Cu^II^-binding in HSA.

## Conclusions

In this study, we investigated Cu^II^-binding of HSA and have demonstrated that in addition to the high-affinity ATCUN motif there are two further Cu^II^-binding sites present in HSA. Using a suite of EPR techniques in combination with site-directed mutagenesis, we were able to identify these sites to involve histidine residues H9 and H288, respectively, which both have, to our knowledge, not been linked to putative HSA Cu^II^-binding sites. Given that both sites were occupied at only two molar equivalents of Cu^II^ present in the samples, we conclude that they must have similar affinities, which are, however, much lower compared to the NTS involving H3. While the latter had been demonstrated to have a square planar CuN_4_ coordination, the characteristic high-field spectral component in CW EPR was not observed for any of the other sites, making H3 the only square planar high affinity site. Further studies will show the effect of fatty acid loading on Cu^II^ binding to HSA, and reveal implications for human disease, as has been shown for altered Zn^II^ dependent coagulation in type 2 diabetes.^7^

## Author contributions

Katrin Ackermann: conceptualisation (supporting), data curation (lead), formal analysis (lead), funding acquisition (supporting), investigation (equal), methodology (equal), writing – original draft (lead), writing – review & editing (equal). Dongmei Wu: conceptualisation (supporting), formal analysis (supporting), investigation (equal), methodology (equal), writing – original draft (supporting), writing – review & editing (equal). Alan J. Stewart: conceptualisation (equal), investigation (supporting), formal analysis (supporting), funding acquisition (equal), methodology (supporting), supervision (equal), writing – original draft (supporting), writing – review & editing (equal). Bela E. Bode: conceptualisation (equal), investigation (supporting), formal analysis (supporting), funding acquisition (equal), methodology (supporting), supervision (equal), writing – original draft (supporting), writing – review & editing (equal).

## Data availability

The research data supporting this publication can be accessed at https://doi.org/10.17630/cf121a40-8ee6-491d-b783-5bf65304cd8d.^46^

## Conflicts of interest

There are no conflicts to declare.

## Supplementary Material

DT-053-D4DT00892H-s001
