# Supplementary material for: EPR spectroscopic characterisation of native CuII-binding sites in human serum albumin
Source: Dalton Trans. 2024 Jul 23;53(32):13529–36. doi: 10.1039/d4dt00892h (PMC11320662; doi:10.1039/d4dt00892h)
Supplement: DT-053-D4DT00892H-s001 [file DT-053-D4DT00892H-s001.pdf]

Supplementary Information (SI) for Dalton Transactions.
This journal is © The Royal Society of Chemistry 2024

Supplementary Information

EPR spectroscopic characterisation of native Cu^{II}-binding sites in human serum albumin

Katrin Ackermann,[a] Dongmei Wu,[b] Alan J. Stewart,[b] Bela E. Bode[a*]*

[a]EaStCHEM School of Chemistry, Biomedical Sciences Research Complex, and Centre of Magnetic Resonance, University of St Andrews, St Andrews, KY16 9ST, Scotland; [b]School of Medicine, Biomedical Sciences Research Complex, and Centre of Magnetic Resonance, University of St Andrews, St Andrews, KY16 9TF, Scotland

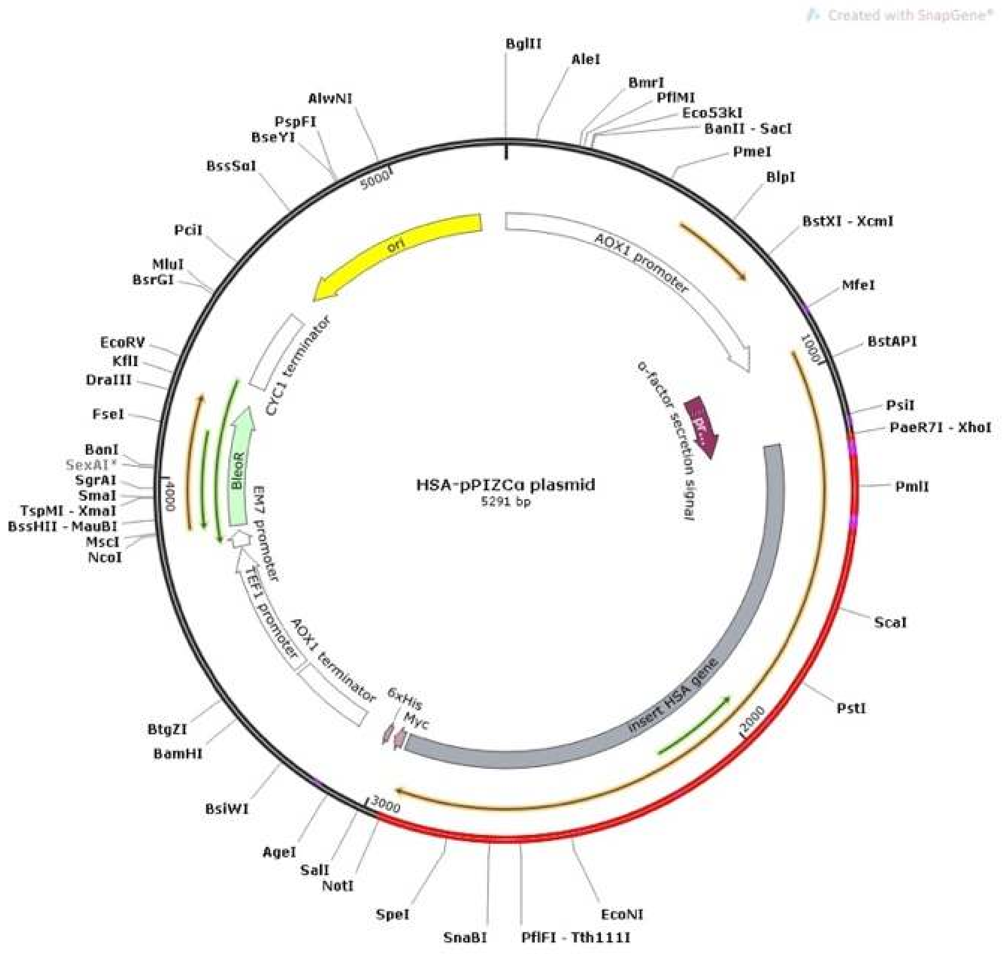

Figure S1. Recombinant HSA-pPICZα plasmid.

The recombinant HSA gene was inserted directly after the region encoding the α-factor secretion signal sequence. The vector map was created using SnapGene software (www.snapgene.com).

Oligo name	Sequence 5′----3′ (length in bases)
His3Ala forward	CTCGAGAAAAGAGATGCTGCCAAGTCTGAAGTCGCTCACAGA (42)
His3Ala reverse	TCTGTGAGCGACTTCAGACTTGGCAGCATCTCTTTTCTCGAG (42)
His9Ala forward	GCTCACAAGTCTGAAGTCGCTGCCAGATTTAAAGACCTTGGT (42)
His9Ala reverse	ACCAAGGTCTTTAAATCTGGCAGCGACTTCAGACTTGTGAGC (42)
His67Ala forward	GAGAATTGCGATAAGTCTCTTGCTACACTTTTCGGCGATAAG (42)
His67Ala reverse	CTTATCGCCGAAAAGTGTAGCAAGAGACTTATCGCAATTCTC (42)
His247Ala forward	GTCCATACTGAATGCTGTGCTGGAGACTTATTGGAATGTGCC (42)
His247Ala reverse	GGCACATTCCAATAAGTCTCCAGCACAGCATTCAGTATGGAC (42)
His288Ala forward	GCCATTGTTGGAAAAGTCCGCTTGCATAGCTGAGGTAGAGAATG (44)
His288Ala reverse	CATTCTCTACCTCAGCTATGCAAGCGGACTTTTCCAACAATGGC (44)
α-factor	TACTATTGCCAGCATTGCTGC
5′ AOX1	GACTGGTTCCAATTGACAAGC
3′ AOX1	GCAAATGGCATTCTGACATCC

Table S1. Primers used for site-directed mutagenesis and DNA sequencing from yeast.

Construct	[albumin] μM	[Cu(II)] μM	Protonated	Deuterated
WT	250	0	X	
WT	250	125 (0.5 eq.)	X	
WT	250	250 (1.0 eq.)	X	
WT	250	375 (1.5 eq.)	X	
WT	250	500 (2.0 eq.)	X	
WT	250	625 (2.5 eq.)	X	
WT	250	750 (3.0 eq.)	X	
WT	250	1000 (4.0 eq.)	X	
WT	250	1250 (5.0 eq.)	X	
WT	250	0		X
WT	250	125 (0.5 eq.)		X
WT	250	250 (1.0 eq.)		X
WT	250	375 (1.5 eq.)		X
WT	250	500 (2.0 eq.)		X
WT	250	625 (2.5 eq.)		X
WT	250	750 (3.0 eq.)		X
WT	250	1000 (4.0 eq.)		X
WT	250	1250 (5.0 eq.)		X
rHSA H3A	241	217 (0.9 eq.)	X	
rHSA WT	250	500 (2.0 eq.)		X
rHSA H3A	250	500 (2.0 eq.)		X
rHSA H9A	250	500 (2.0 eq.)		X
rHSA H67A	250	500 (2.0 eq.)		X
rHSA H247A	250	500 (2.0 eq.)		X
rHSA H288A	250	500 (2.0 eq.)		X

Table S2. Detailed sample list for EPR measurements. WT = wild-type (plasma-purified); rHSA = recombinant human serum albumin; all mutant HSA variants were produced recombinantly.

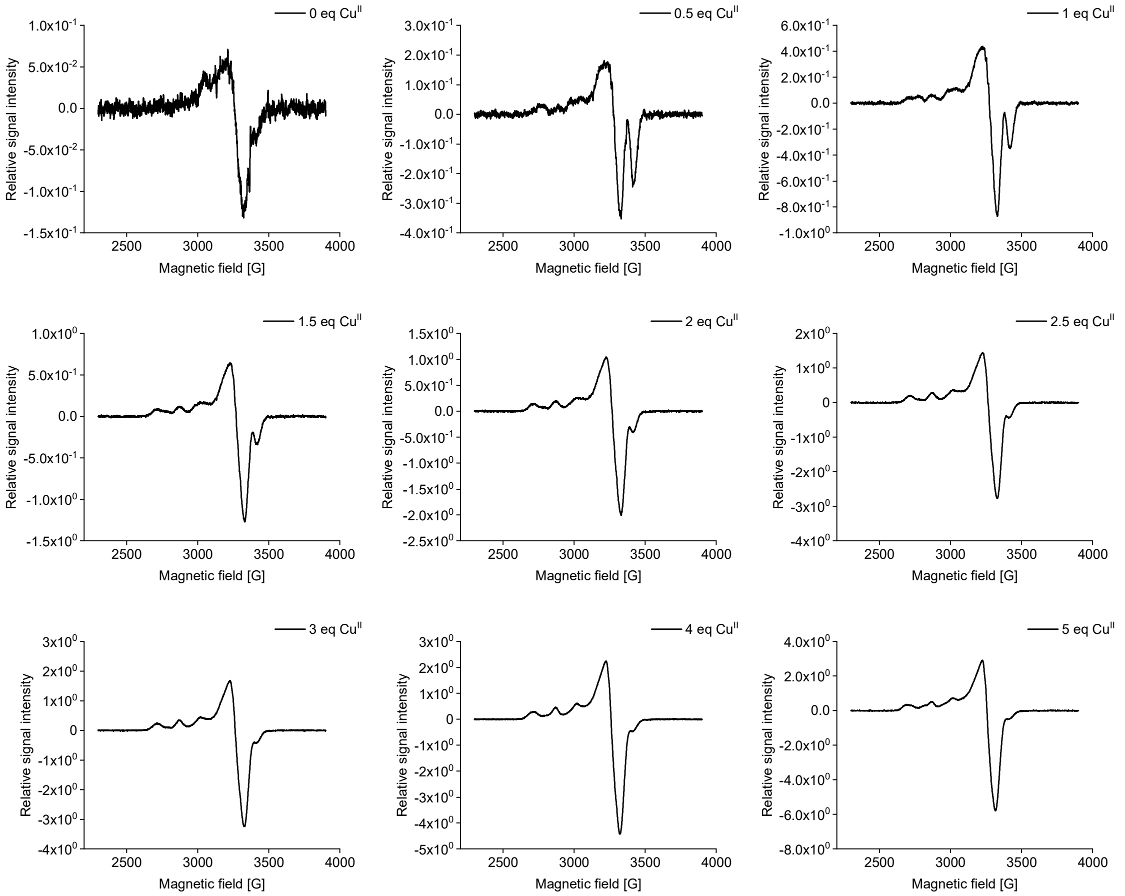

Figure S2. Individual CW EPR spectra of the HSA pseudo-titration series with 0 to 5 molar equivalents of Cu^{II} added.

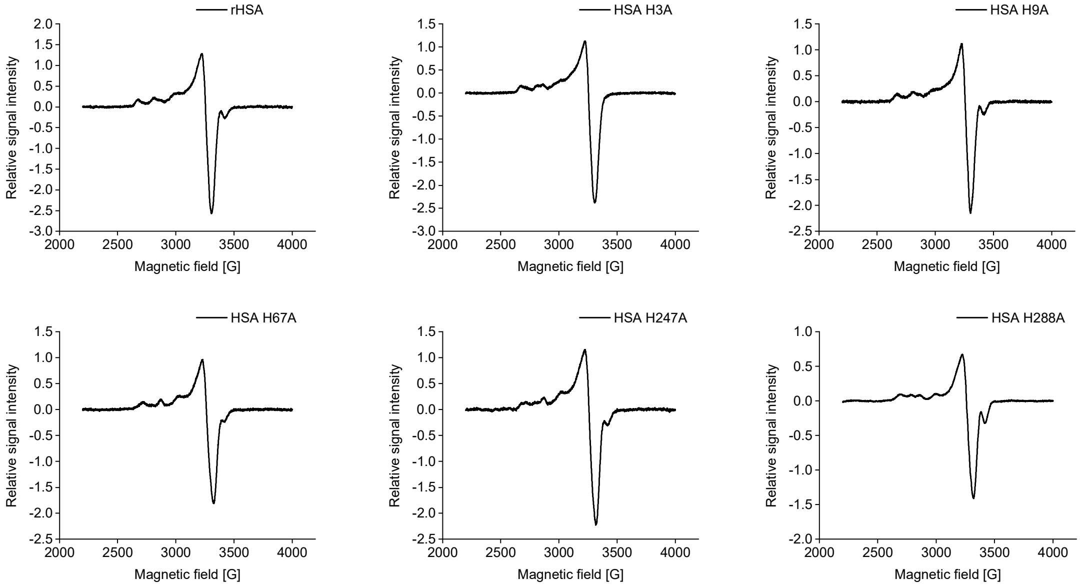

Figure S3. Individual CW EPR spectra of the recombinant HSA series with 2 molar equivalents of Cu^{II} added. The respective mutant variant is indicated on each plot. Note the absence of the H3 spectral feature at ~3420 G in the H3A mutant. rHSA = wild-type recombinant HSA.

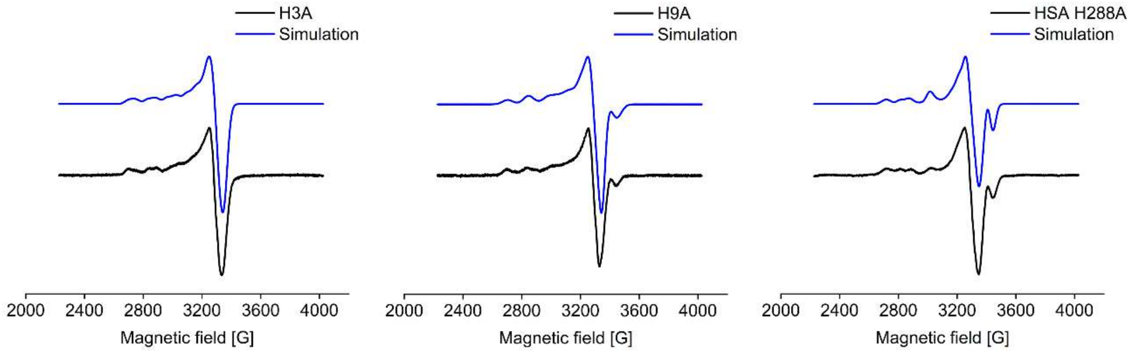

Figure S4. Experimental (black) and simulated (blue) CW spectra for the H3A (left), H9A (middle) and H288A (right) mutant of HSA. Corresponding simulation parameters are given below in **Table S3**.

**Spectrum**	**H3A**		**H288A**		**H9A**	
**Component**	**1**	**2**	**1**	**2**	**1**	**2**
**Centre**	**H288**	**H9**	**H9**	**H3**	**H288**	**H3**
g_{11}	2.0447 (31)	2.0486 (136)	2.0558 (25)	2.0391 (87)	2.0527 (77)	2.0385 (47)
g_{22}	2.0602 (48)	2.0844 (21)	2.0560 (25)	2.0851 (105)	2.0636 (97)	2.1121 (186)
g_{33}	2.3420 (17)	2.2979 (38)	2.3063 (75)	2.1840 (150)	2.3341 (520)	2.1647 (59)
A_{11}	43 (19)	7 (109)	16 (51)	8 (75)	43 (33)	8 (42)
A_{22}	25 (35)	8 (99)	15 (50)	7 (98)	27 (30)	7 (121)
A_{33}	424 (46)	430 (13)	483 (50)	605 (50)	438 (14)	556 (26)
Linewidth	5.6	5.97	6.9	4.0	4.84	8.2
Weight	0.514	0.486	0.73	0.27	0.70	0.30

Table S3. CW EPR simulation parameters for spectra shown in **Figure S4** above. A values are given in MHz and rounded, values for the linewidth are given in mT, the numbers in brackets are the strain corresponding to the inhomogeneous broadening for the respective parameter. A and g values were anti-correlated (-1) for all components.

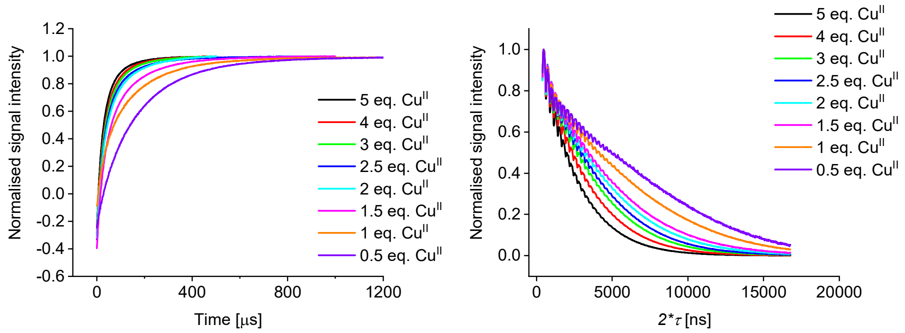

Figure S5. Three-pulse inversion recovery experiments (left) and two-pulse echo decay experiments (right) for samples of the pseudo-titration series to estimate longitudinal relaxation times and transverse dephasing times, respectively (see Table S3).

Eq. Cu^{II}	T_1	T_1	1/e time	½ × 1/e^2 time	T_2	1/e time	½ × 1/e^2 time
	τ_1, μs	τ_2, μs	μs	μs	μs	μs	μs
0.5	216	78	72	107	18.9	4.1	3.3
1.0	182	39	32	81	3.7	3.4	2.9
1.5	138	30	33	61	2.5	2.8	2.4
2.0	105	26	25	44	2.3	2.6	2.2
2.5	111	27	23	39	2.1	2.4	2.0
3.0	84	23	21	35	1.9	2.2	1.8
4.0	77	22	20	32	1.6	1.9	1.6
5.0	67	19	16	28	1.2	1.5	1.4

Table S4. Estimated T_1 values under the biexponential approximation and T_2 (~T_m) values under the stretched exponential approximation, including respective 1/e and ½ × 1/e^2 times. Note the substantial difference in relaxation times between the 0.5 molar equivalents of Cu^{II} and the higher Cu^{II} load samples.

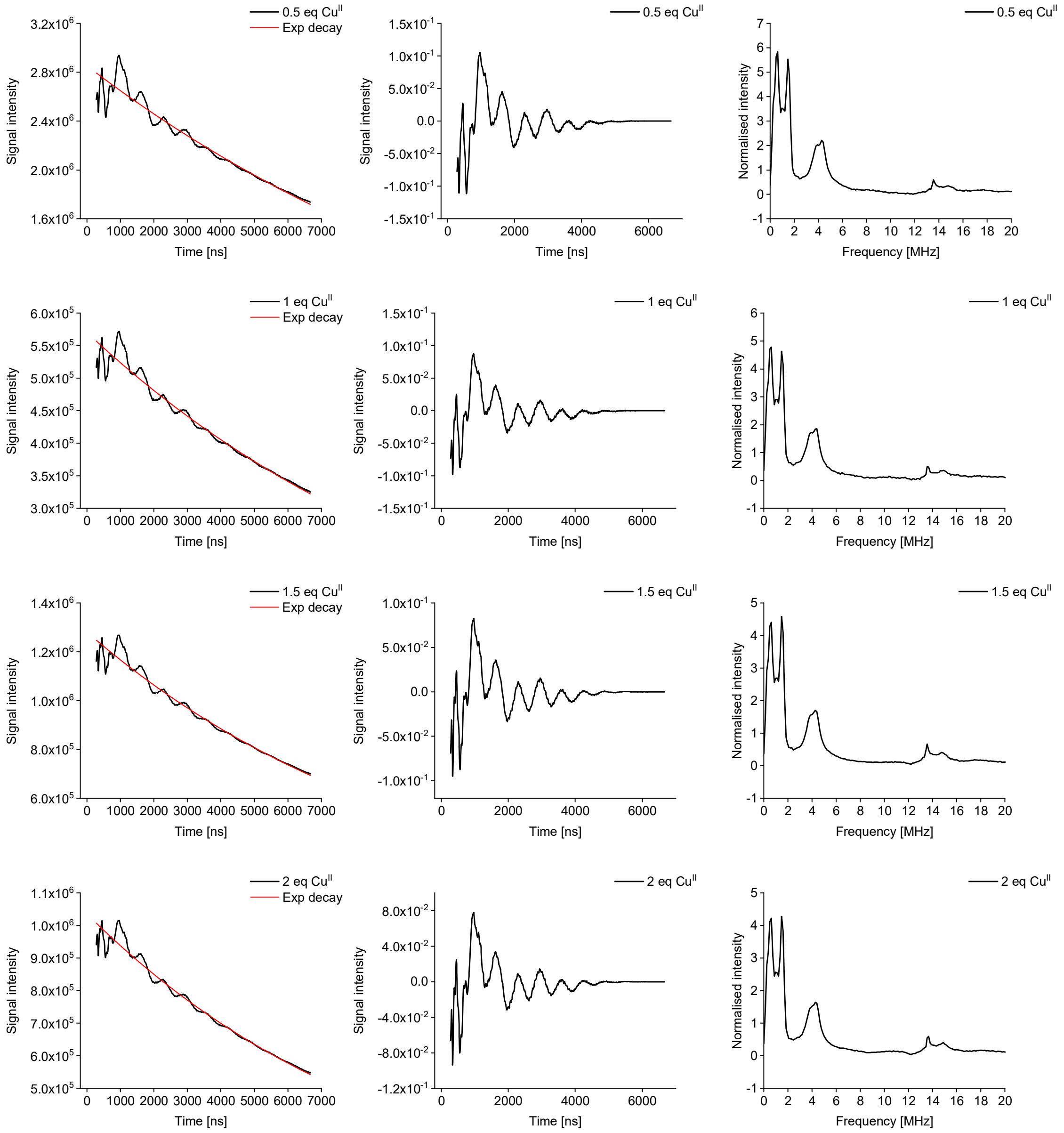

Figure S6. Individual ESEEM spectra of the pseudo-titration series with 0.5 to 5.0 molar equivalents of Cu^{II} added. Shown are the raw ESEEM traces (black) with fitted exponential background decay (red, left), the background-corrected traces (middle), and the corresponding absolute (magnitude) spectra after FFT (right).

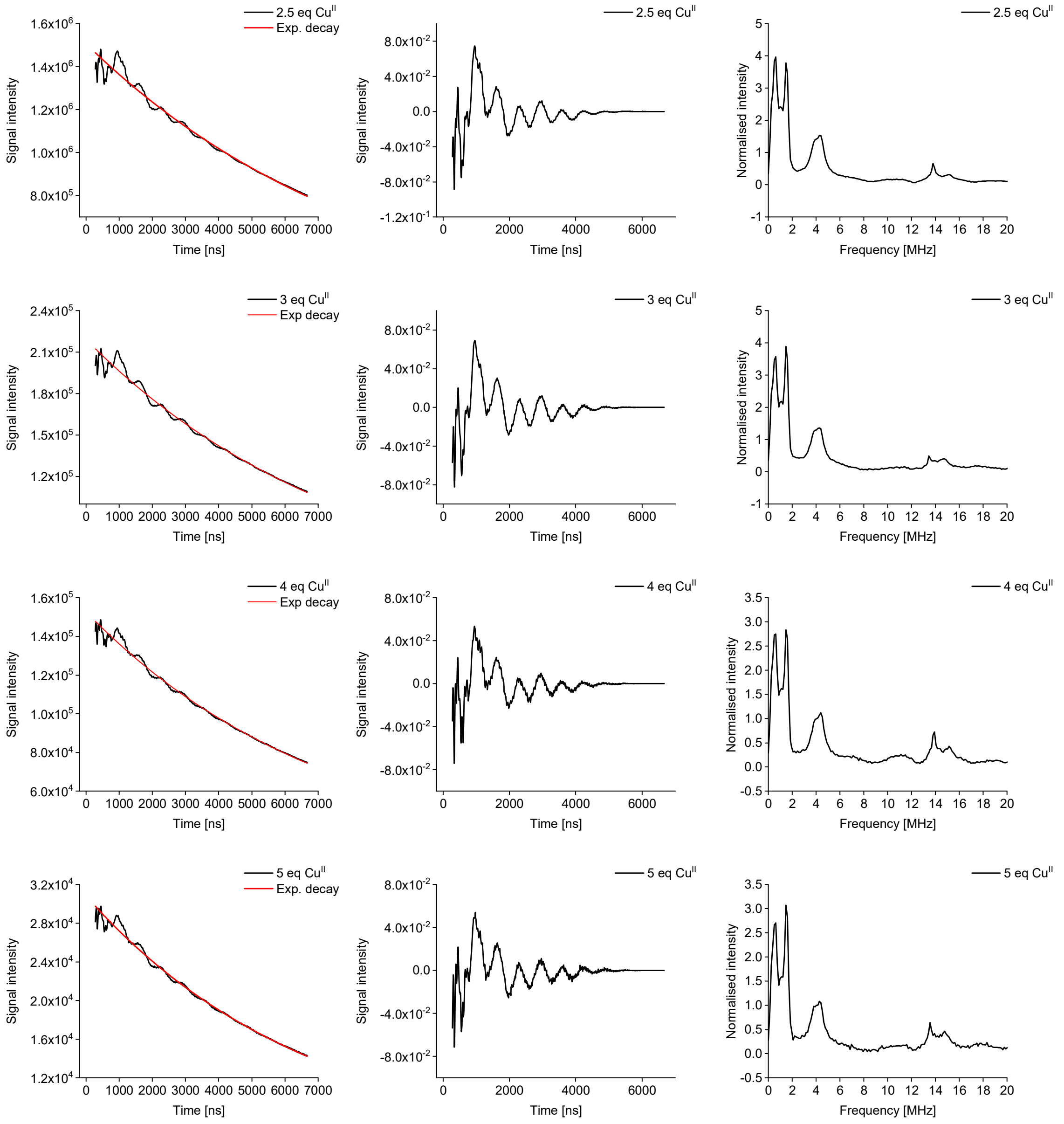

Figure S6, continued. Individual ESEEM spectra of the pseudo-titration series with 0.5 to 5.0 molar equivalents of Cu^{II} added. Shown are the raw ESEEM traces (black) with fitted exponential background decay (red, left), the background-corrected traces (middle), and the corresponding absolute (magnitude) spectra after FFT (right).

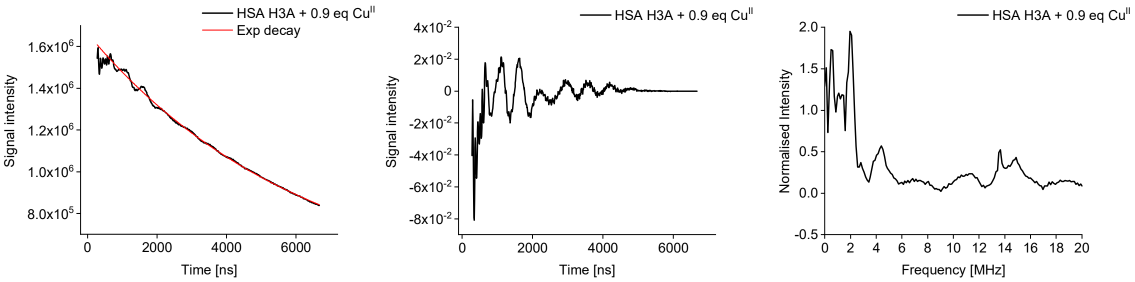

Figure S7. ESEEM spectrum of the protonated HSA H3A mutant with 0.9 molar equivalents of Cu^{II} added. NQI and DQ peaks are clearly visible, indicating a histidine environment for the Cu^{II} coordination. Shown is the raw ESEEM trace (black) with fitted exponential background decay (red, left), the background-corrected trace (middle), and the corresponding absolute (magnitude) spectrum after FFT (right).

HYSCORE with 0.5 molar equivalents of Cu^{II}:

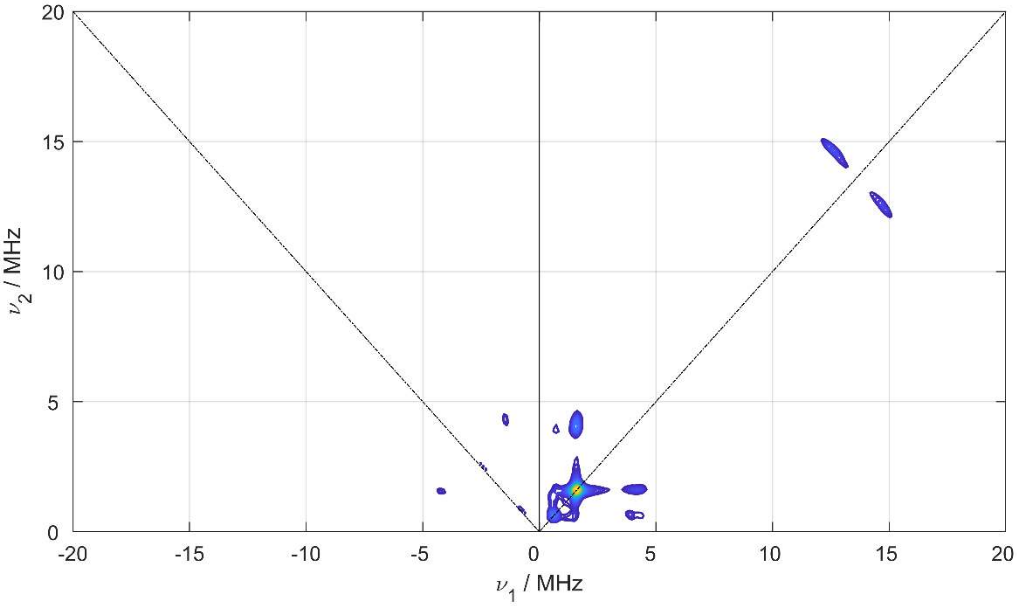

Figure S8. Individual HYSCORE spectra of the pseudo-titration series. From top to bottom: 0.5 / 1.0 / 1.5 / 2.0 / 2.5 / 3.0 / 4.0 / 5.0 molar equivalents of Cu^{II}.

HYSCORE with 1.0 molar equivalent of Cu^{II}:

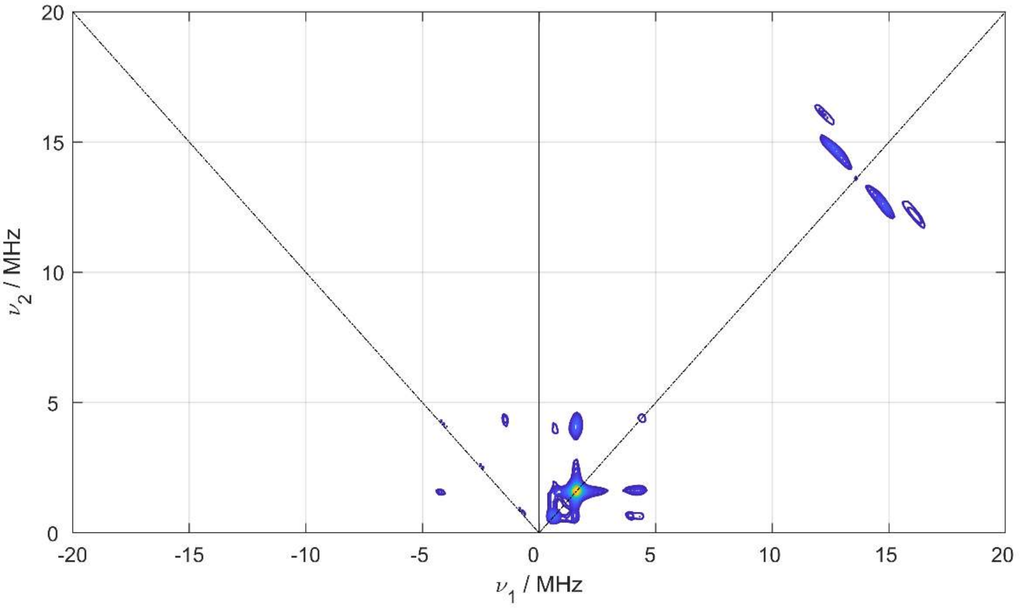

HYSCORE with 1.5 molar equivalent of Cu^{II}:

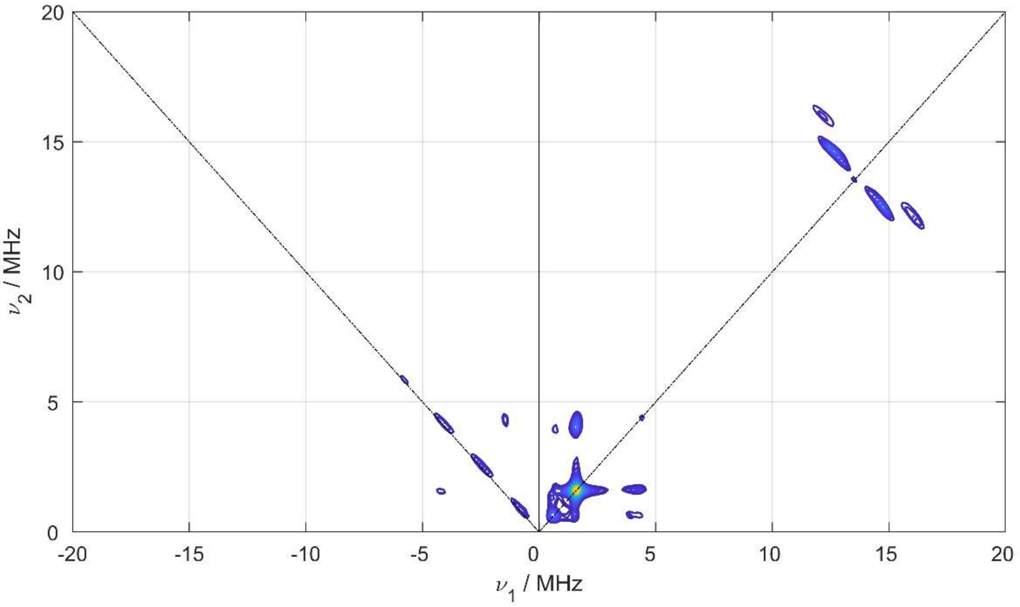

Figure S8, continued. Individual HYSCORE spectra of the pseudo-titration series. From top to bottom: 0.5 / 1.0 / 1.5 / 2.0 / 2.5 / 3.0 / 4.0 / 5.0 molar equivalents of Cu^{II}.

HYSCORE with 2.0 molar equivalent of Cu^{II}:

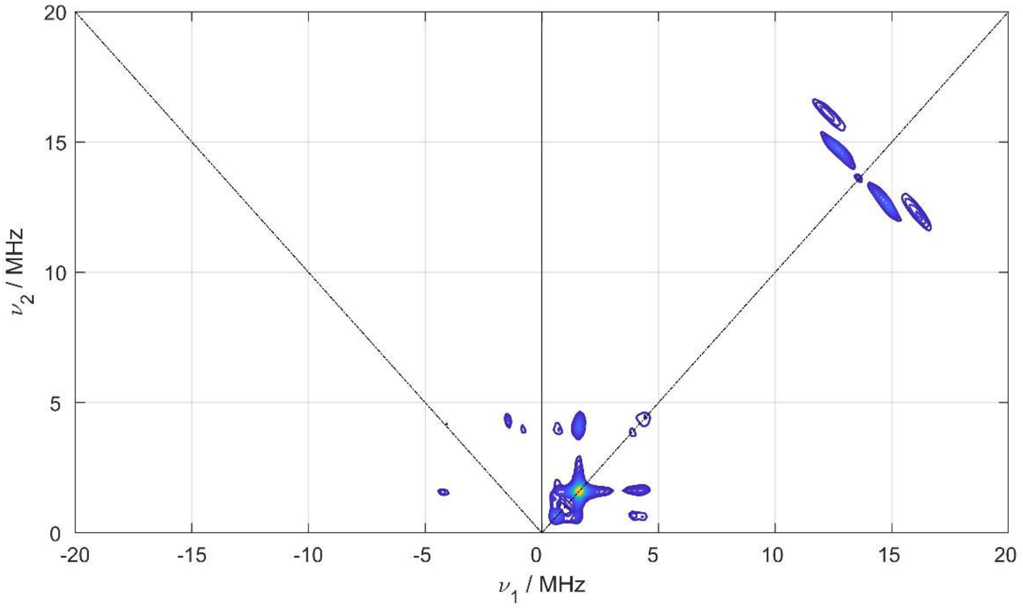

HYSCORE with 2.5 molar equivalent of Cu^{II}:

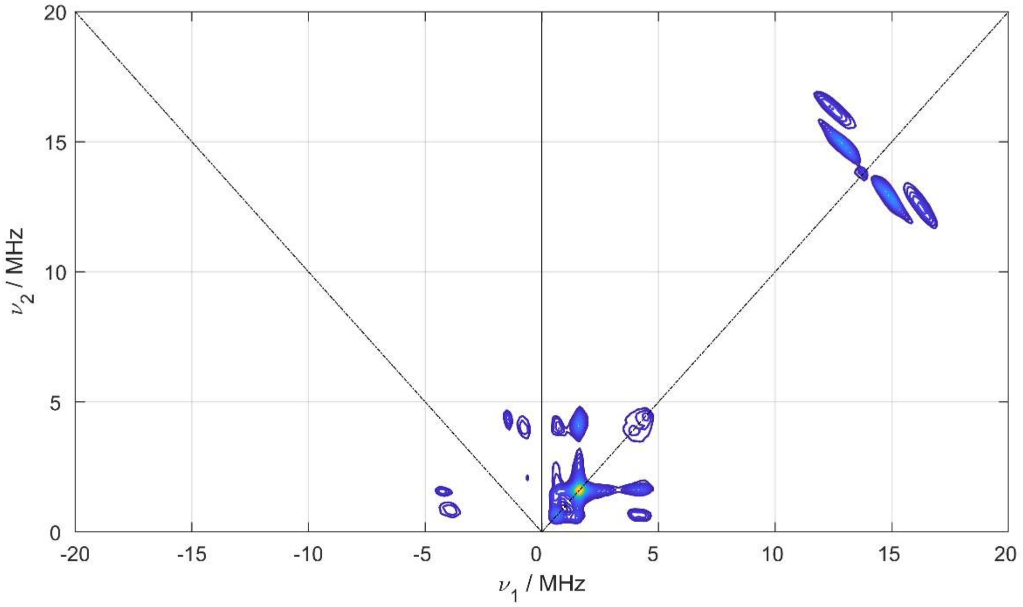

Figure S8, continued. Individual HYSCORE spectra of the pseudo-titration series. From top to bottom: 0.5 / 1.0 / 1.5 / 2.0 / 2.5 / 3.0 / 4.0 / 5.0 molar equivalents of Cu^{II}.

HYSCORE with 3.0 molar equivalent of Cu^{II}:

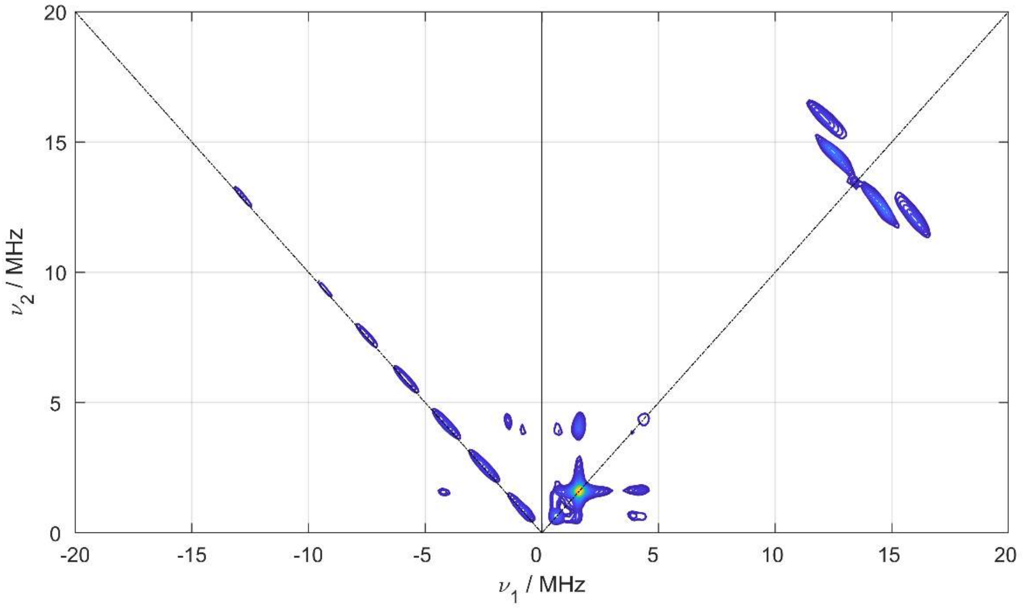

HYSCORE with 4.0 molar equivalent of Cu^{II}:

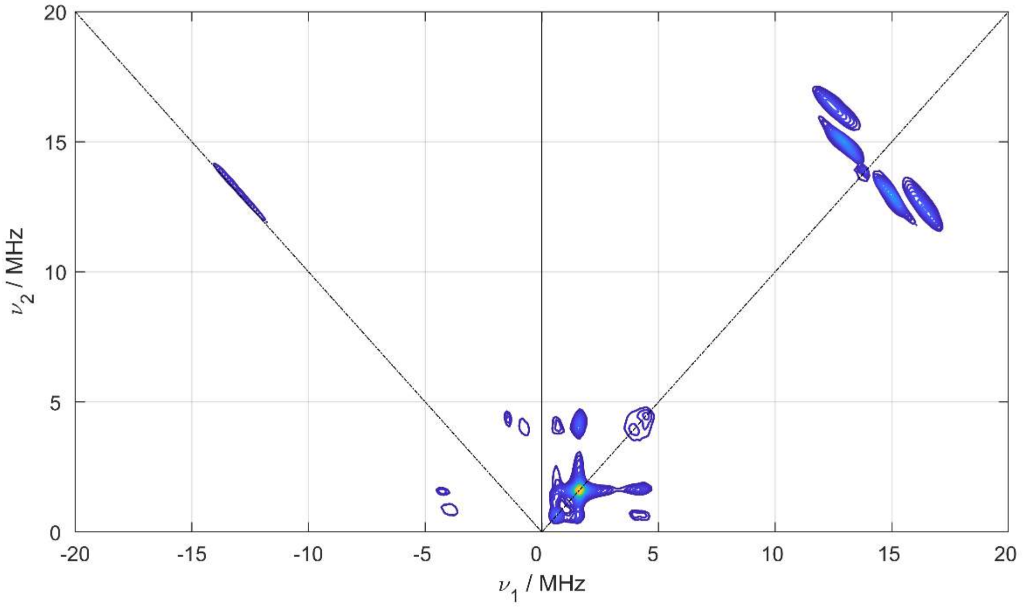

Figure S8, continued. Individual HYSCORE spectra of the pseudo-titration series. From top to bottom: 0.5 / 1.0 / 1.5 / 2.0 / 2.5 / 3.0 / 4.0 / 5.0 molar equivalents of Cu^{II}.

HYSCORE with 5.0 molar equivalent of Cu^{II}:

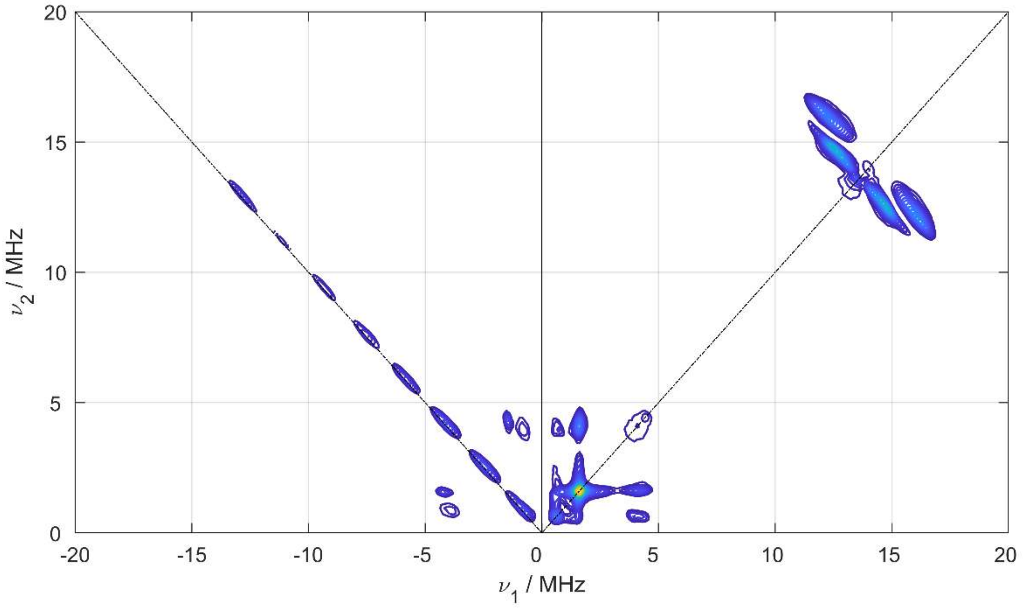

Figure S8, continued. Individual HYSCORE spectra of the pseudo-titration series. From top to bottom: 0.5 / 1.0 / 1.5 / 2.0 / 2.5 / 3.0 / 4.0 / 5.0 molar equivalents of Cu^{II}.

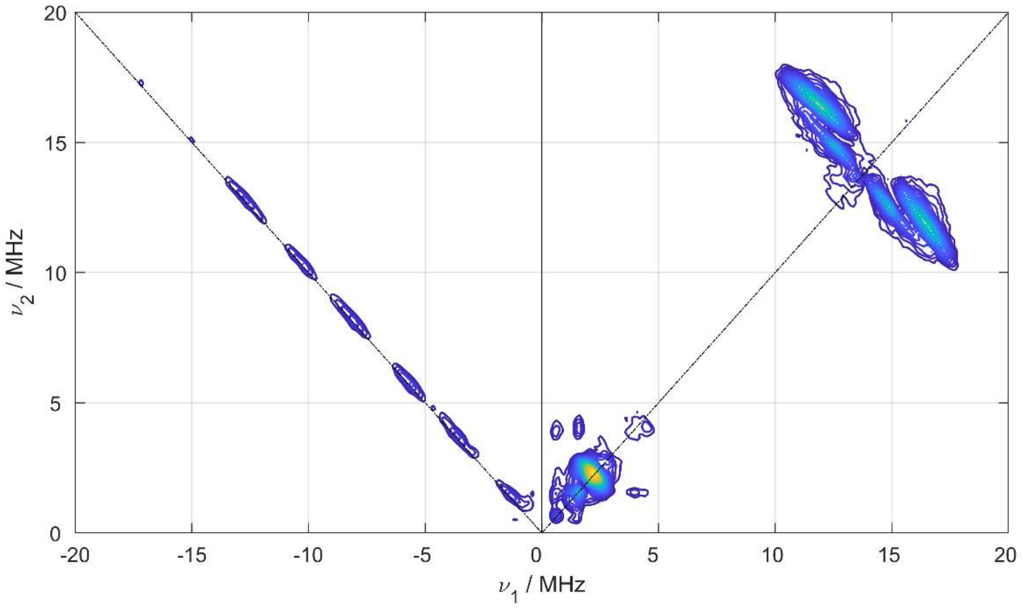

Figure S9. HYSCORE spectrum of the protonated HSA H3A mutant with 0.9 molar equivalents of Cu^{II} added.

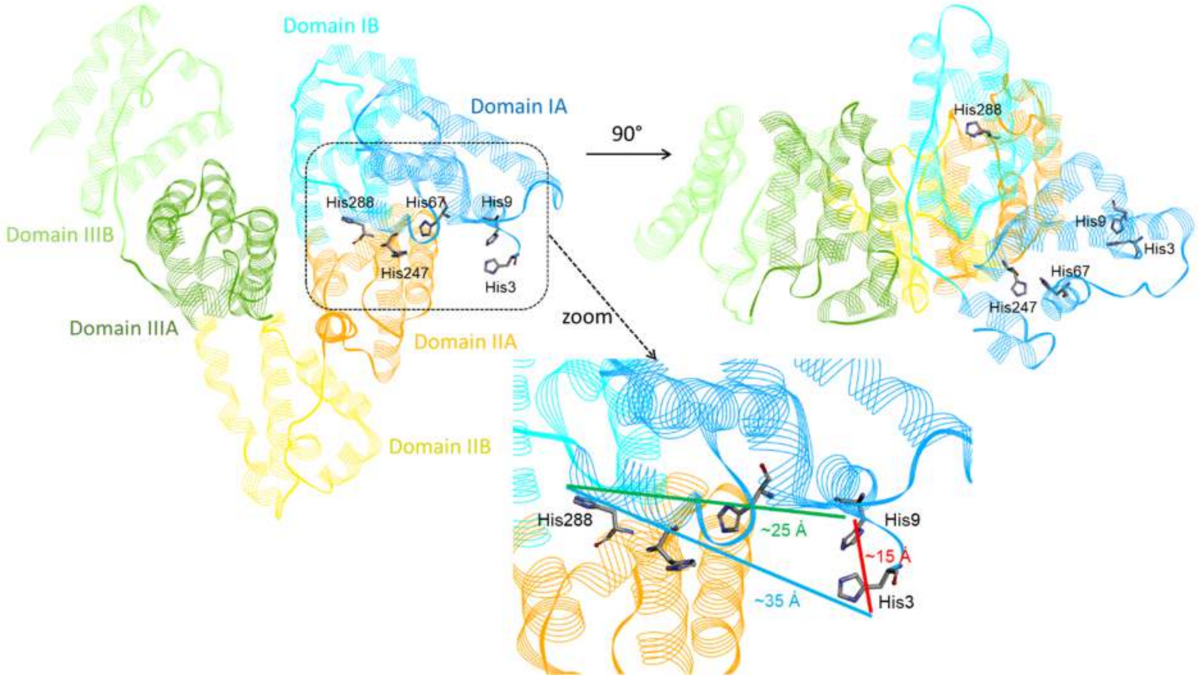

Figure S10. Structure of HSA showing locations of studied histidine residues. Individual sub-domains are highlighted by colour. The structure shown on the right is rotated by 90 degrees relative to the structure on the left, providing the view from above. Cartoons were drawn from structural coordinates obtained from the RSCB Protein Data Bank (PDB ID: 5IJF). Note that the sidechains of His3 and His9 were partially missing in the x-ray structure and were modelled using Discovery Studio Visualizer v24.1.0.23298 (Dassault Systèmes Biova Corp.). The inset shows the enlarged histidine-triangle involved in Cu^{II}-binding of HSA, with His3 as primary and His9 and His288 as secondary sites, based on affinity; approximate distances between the sites as estimated from RIDME measurements are indicated.

Sample	CDA2.0 report name
rHSA WT	230215_KAq205.3_RIDME_20_50_50000_comparative_DEER_analyzer_report
rHSA H3A	230215_KAq205.6_RIDME_18_36_36000_comparative_DEER_analyzer_report
rHSA H9A	230216_KAq205.9_RIDME_28_67_67000_comparative_DEER_analyzer_report
rHSA H67A	230217_KAq205.15_RIDME_28_57_57000_comparative_DEER_analyzer_report
rHSA H247A	230216_KAq205.12_RIDME_26_55_55000_comparative_DEER_analyzer_report
rHSA H288A	230618_BEBQ82.6_RIDME_comparative_DEER_analyzer_report

Table S5. CDA2.0 report names for RIDME measurements, see appendix for full reports.

DEER analysis report on dataset 230215_KAq205.3_RIDME_20_50_50000

DEERNet Spinach SVN Rev 5662 and DeerLab 0.9.1 Tikhonov regularization

ComparativeDEERAnalyzer version 2.0

see: S. G. Worswick et al., DOI: 10.1126/sciadv.aat5218, L. Fabregas Ibanez et al., DOI: 10.5194/mr-1-209-2020

02-Mar-2023 14:04:46

1. Distance distributions

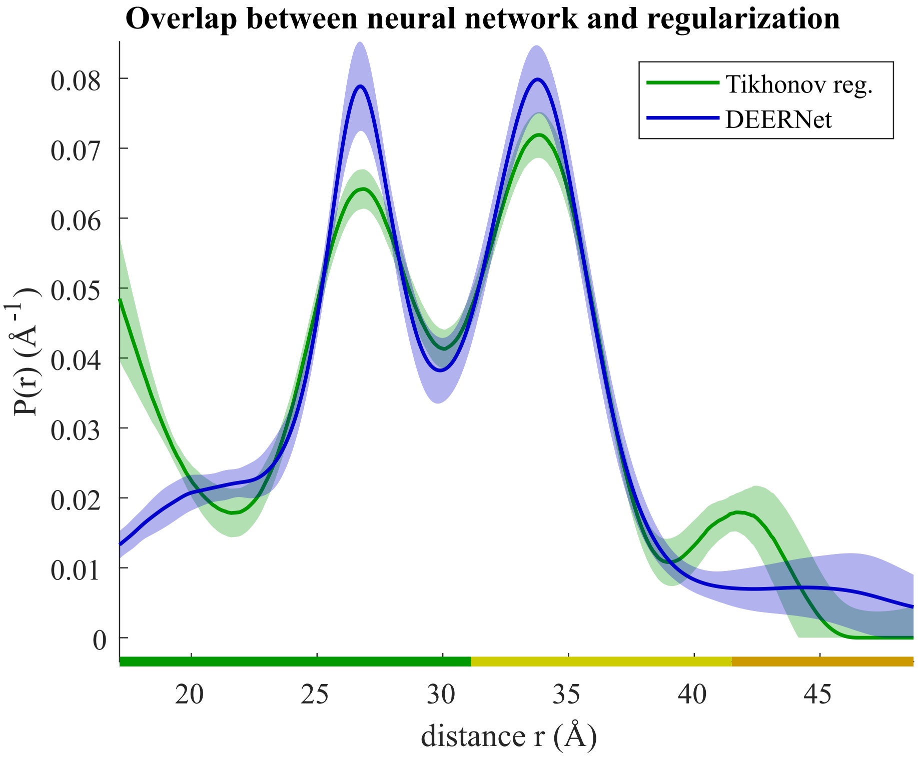

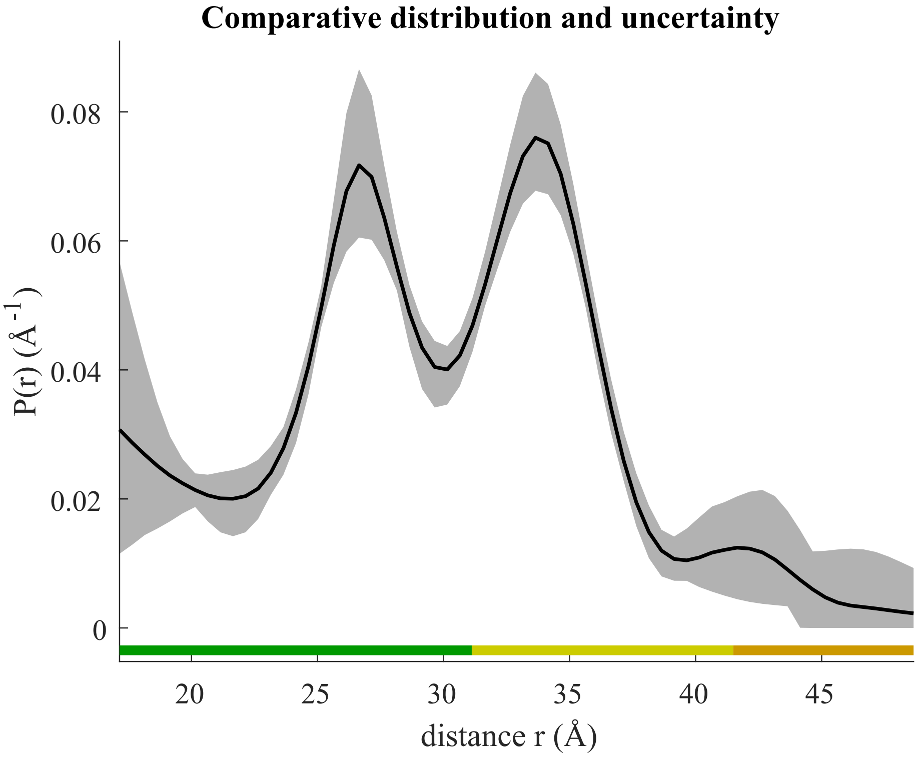

2. Fits of time-domain data

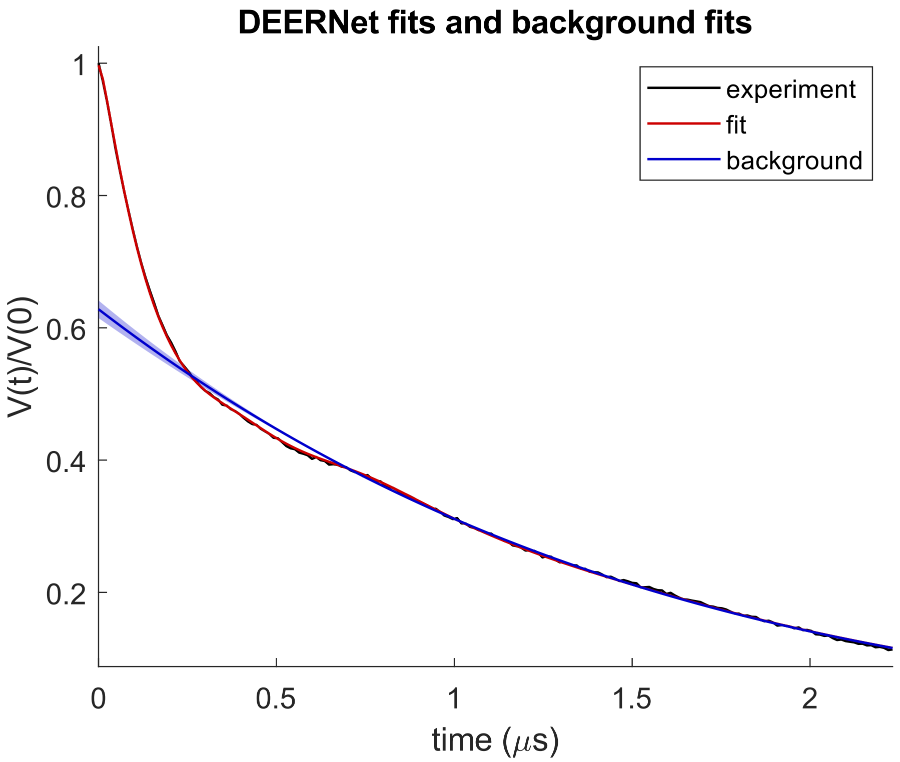

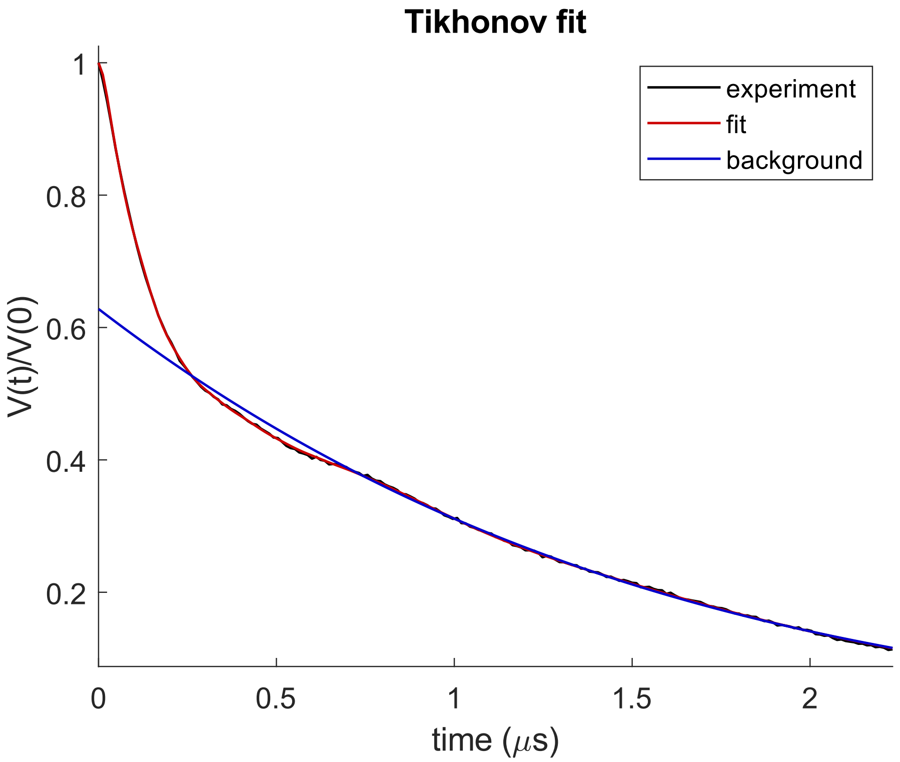

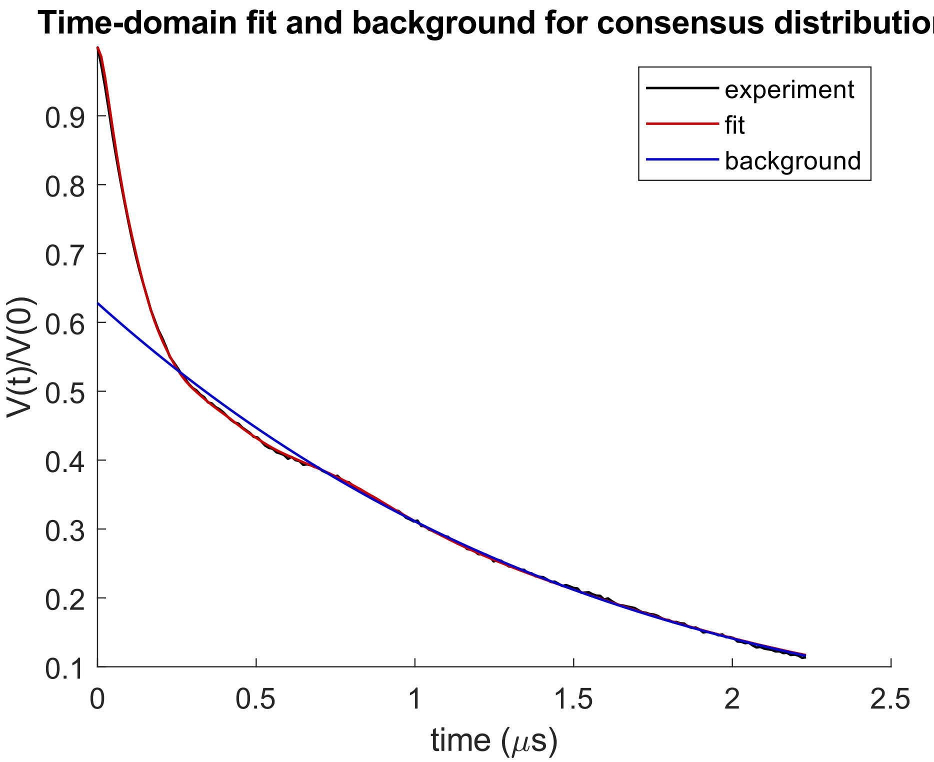
Time-domain fit and background for consensus distribution
experiment
fit
background
V(t)/V(0)
0.9
0.8
0.7
0.6
0.5
0.4
0.3
0.2
0.1
0
0.5
1
1.5
2
2.5
time (μs)

3. Experimental and processing parameters

RIDME processing was requested. Only DEERNet output.

Modulation depth: 0.370

Signal-to-noise ratio: 188.1 (w.r.t. modulation)

Noise estimates normalized to maximum signal

From imaginary part: 0.00000

From DEERNet fit: 0.00197

From Tikhonov fit: 0.00217

Zero time: 8 ns

Maximum time: 2232 ns

Time increment: 12 ns

Phase: 0.0 degree

Ensemble of 32 neural networks

Background separation by neural network

Background dimension: 3

Regularization parameter by best overlap with neural network solution

Regularization parameter used: 2.24

Reg. par. initial estimate by L-curve corner: 1.58

Overlap between DEERNet and regularization solutions: 0.909

Predicted overlap of consensus solution with ground truth: 0.78...0.95

Mean distance: 29.5 Å

Distance standard deviation: 7.1 Å

Full data set in Matlab format: C:\Users\ka44\Documents\OneDrive - University of St Andrews\StAndrews\Work\BEB\Projects\Albumin\HSA_EPR\HSA_Q\KAq205\205\230215_KAq205.3_RIDME_20_50_50000_comparative_DEER_analysis.mat

Distance distributions in text format: C:\Users\ka44\Documents\OneDrive - University of St Andrews\StAndrews\Work\BEB\Projects\Albumin\HSA_EPR\HSA_Q\KAq205\205\230215_KAq205.3_RIDME_20_50_50000_consensus_DEER_distribution.csv

Fit and background in text format: C:\Users\ka44\Documents\OneDrive - University of St Andrews\StAndrews\Work\BEB\Projects\Albumin\HSA_EPR\HSA_Q\KAq205\205\230215_KAq205.3_RIDME_20_50_50000_consensus_DEER_fit.csv

Metadata: C:\Users\ka44\Documents\OneDrive - University of St Andrews\StAndrews\Work\BEB\Projects\Albumin\HSA_EPR\HSA_Q\KAq205\205\230215_KAq205.3_RIDME_20_50_50000_comparative_DEER_meta_data.csv

DEER analysis report on dataset 230215_KAq205.6_RIDME_18_36_36000

DEERNet Spinach SVN Rev 5662 and DeerLab 0.9.1 Tikhonov regularization

ComparativeDEERAnalyzer version 2.0

see: S. G. Worswick et al., DOI: 10.1126/sciadv.aat5218, L. Fabregas Ibanez et al., DOI: 10.5194/mr-1-209-2020

02-Mar-2023 14:08:09

1. Distance distributions

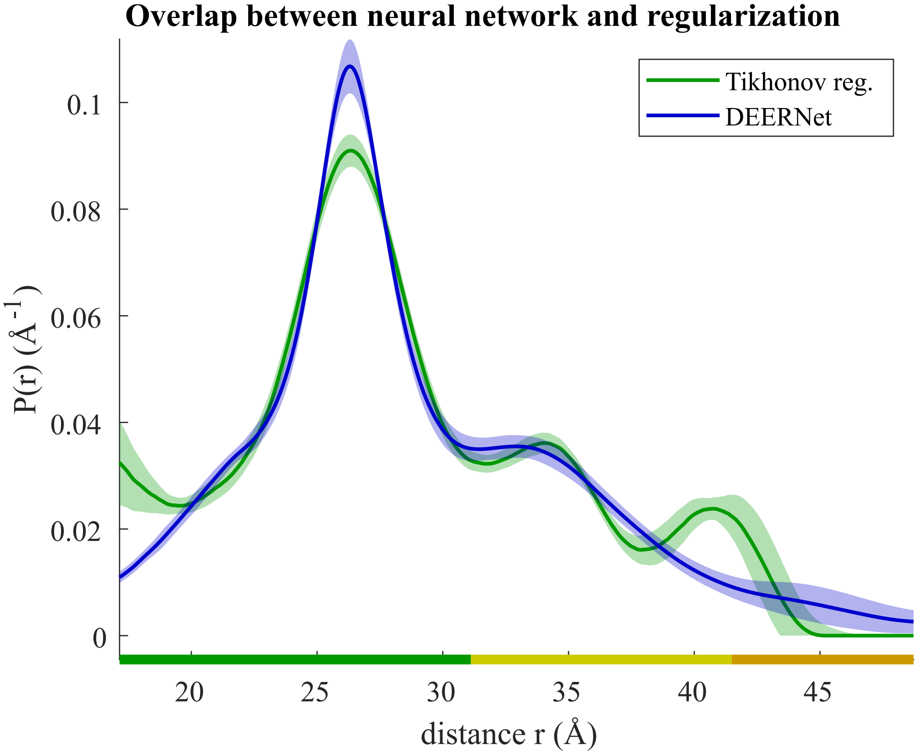

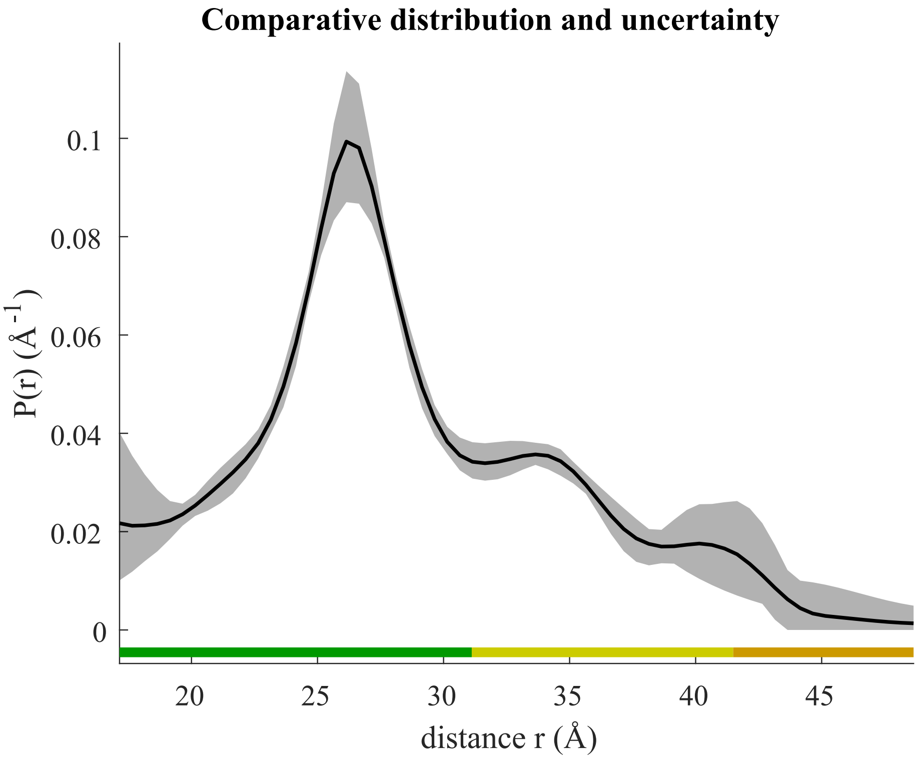

2. Fits of time-domain data

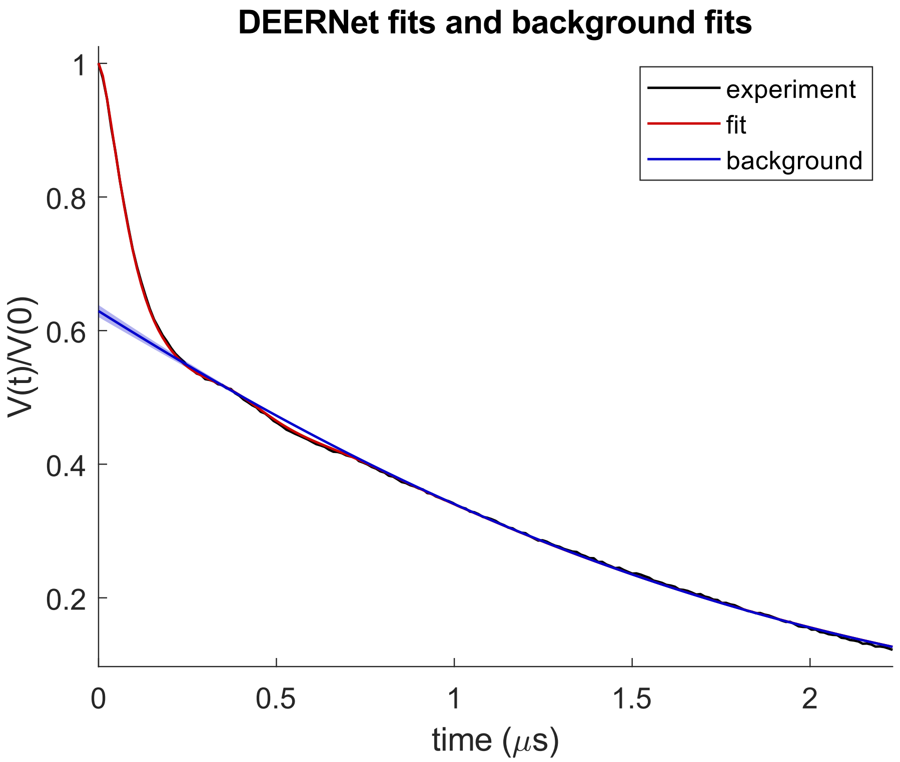

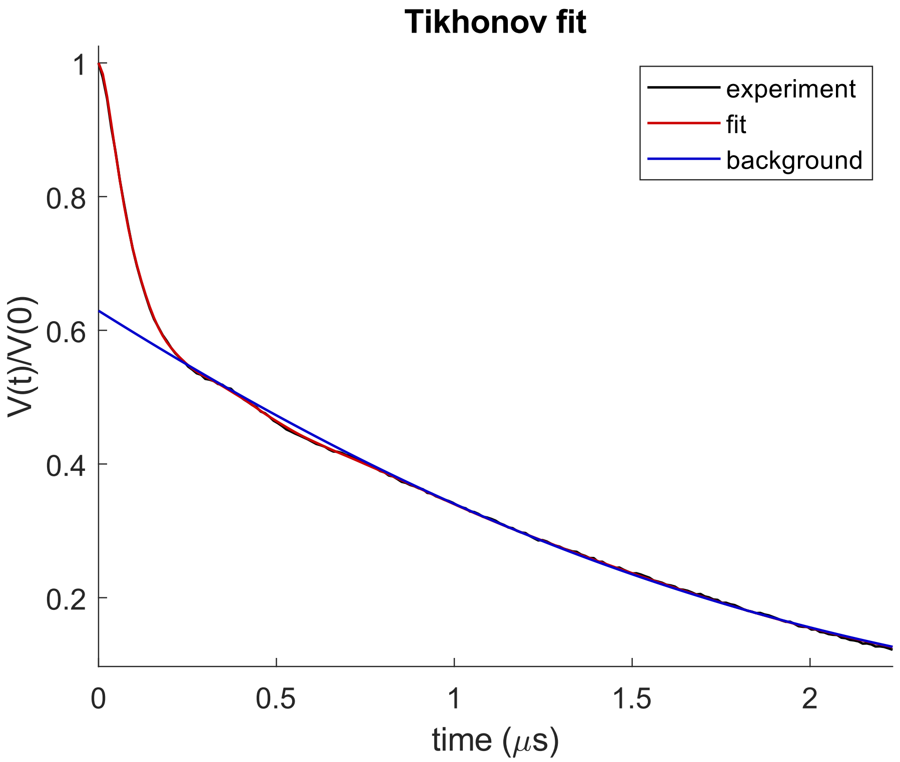

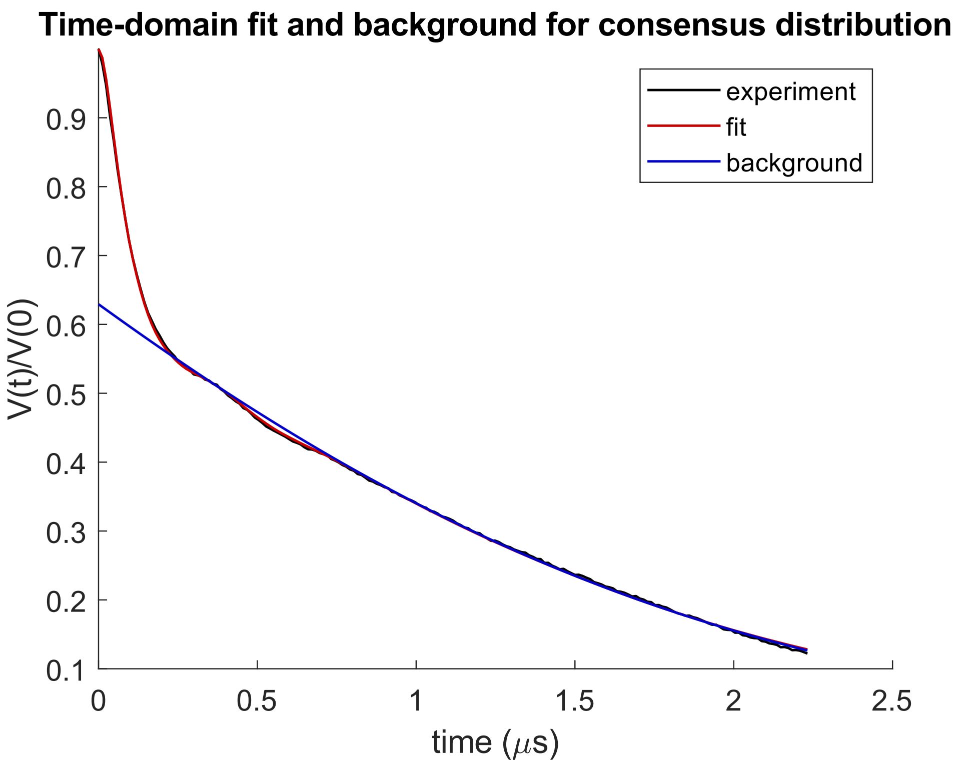

Time-domain fit and background for consensus distribution
experiment
fit
background
V(t)/V(0)
0.1
0.2
0.3
0.4
0.5
0.6
0.7
0.8
0.9
0
0.5
1
1.5
2
2.5
time (μs)

3. Experimental and processing parameters

RIDME processing was requested. Only DEERNet output.

Modulation depth: 0.371

Signal-to-noise ratio: 165.7 (w.r.t. modulation)

Noise estimates normalized to maximum signal

From imaginary part: 0.00000

From DEERNet fit: 0.00224

From Tikhonov fit: 0.00231

Zero time: 8 ns

Maximum time: 2232 ns

Time increment: 12 ns

Phase: 0.0 degree

Ensemble of 32 neural networks

Background separation by neural network

Background dimension: 3

Regularization parameter by best overlap with neural network solution

Regularization parameter used: 2.00

Reg. par. initial estimate by L-curve corner: 1.00

Overlap between DEERNet and regularization solutions: 0.921

Predicted overlap of consensus solution with ground truth: 0.79...0.96

Mean distance: 26.9 Å

Distance standard deviation: 8.0 Å

Full data set in Matlab format: C:\Users\ka44\Documents\OneDrive - University of St Andrews\StAndrews\Work\BEB\Projects\Albumin\HSA_EPR\HSA_Q\KAq205\205\230215_KAq205.6_RIDME_18_36_36000_comparative_DEER_analysis.mat

Distance distributions in text format: C:\Users\ka44\Documents\OneDrive - University of St Andrews\StAndrews\Work\BEB\Projects\Albumin\HSA_EPR\HSA_Q\KAq205\205\230215_KAq205.6_RIDME_18_36_36000_consensus_DEER_distribution.csv

Fit and background in text format: C:\Users\ka44\Documents\OneDrive - University of St Andrews\StAndrews\Work\BEB\Projects\Albumin\HSA_EPR\HSA_Q\KAq205\205\230215_KAq205.6_RIDME_18_36_36000_consensus_DEER_fit.csv

Metadata: C:\Users\ka44\Documents\OneDrive - University of St Andrews\StAndrews\Work\BEB\Projects\Albumin\HSA_EPR\HSA_Q\KAq205\205\230215_KAq205.6_RIDME_18_36_36000_comparative_DEER_meta_data.csv

DEER analysis report on dataset 230216_KAq205.9_RIDME_28_67_67000

DEERNet Spinach SVN Rev 5662 and DeerLab 0.9.1 Tikhonov regularization

ComparativeDEERAnalyzer version 2.0

see: S. G. Worswick et al., DOI: 10.1126/sciadv.aat5218, L. Fabregas Ibanez et al., DOI: 10.5194/mr-1-209-2020

02-Mar-2023 14:11:32

1. Distance distributions

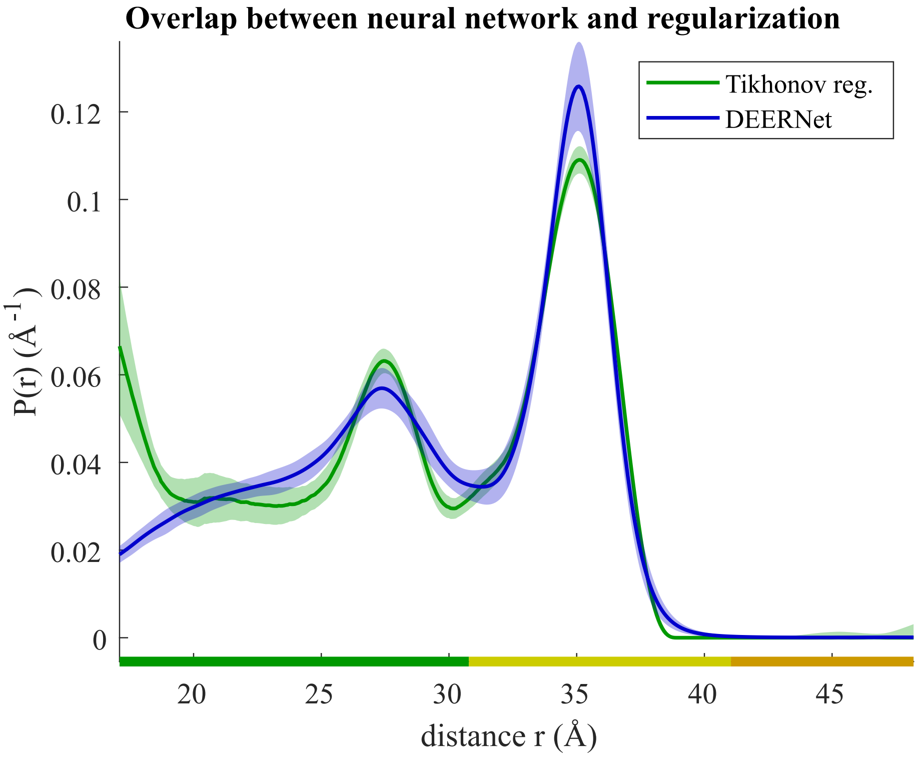

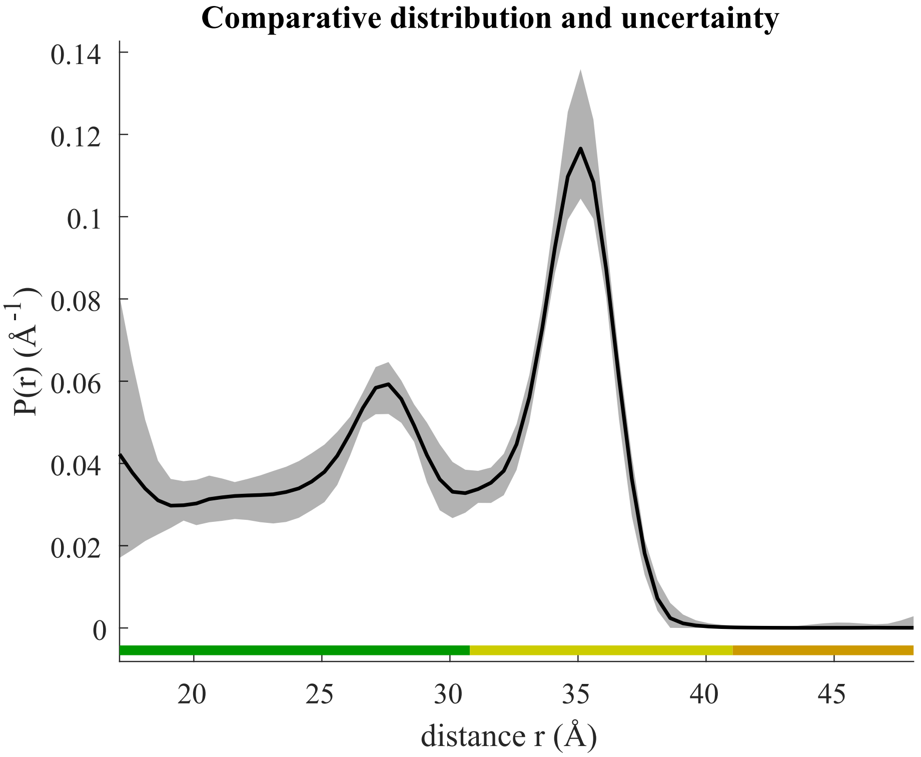

2. Fits of time-domain data

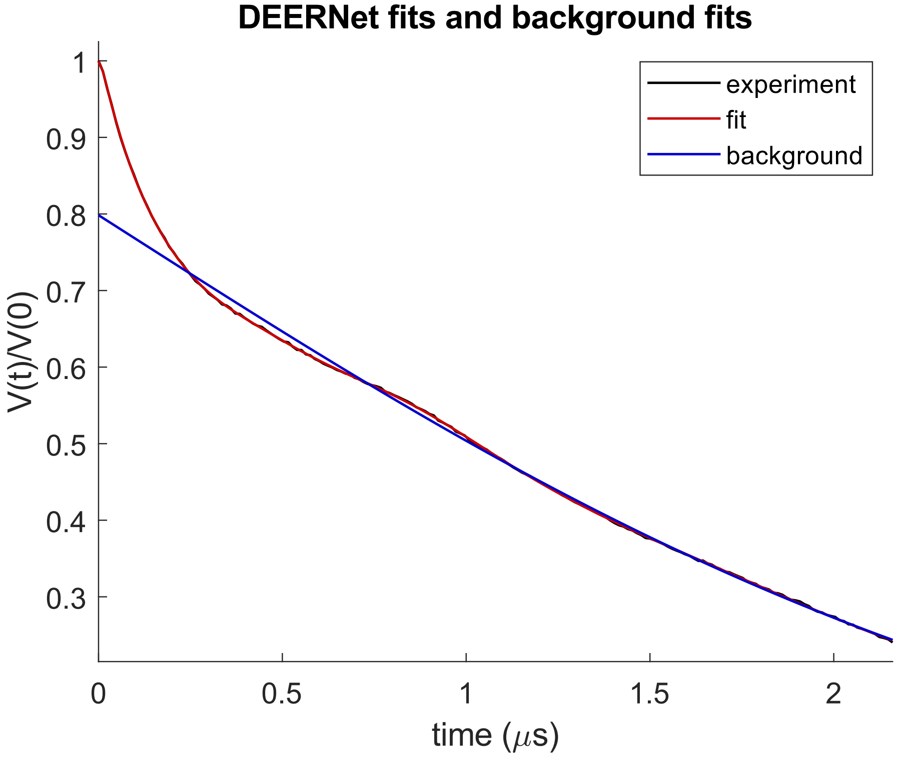

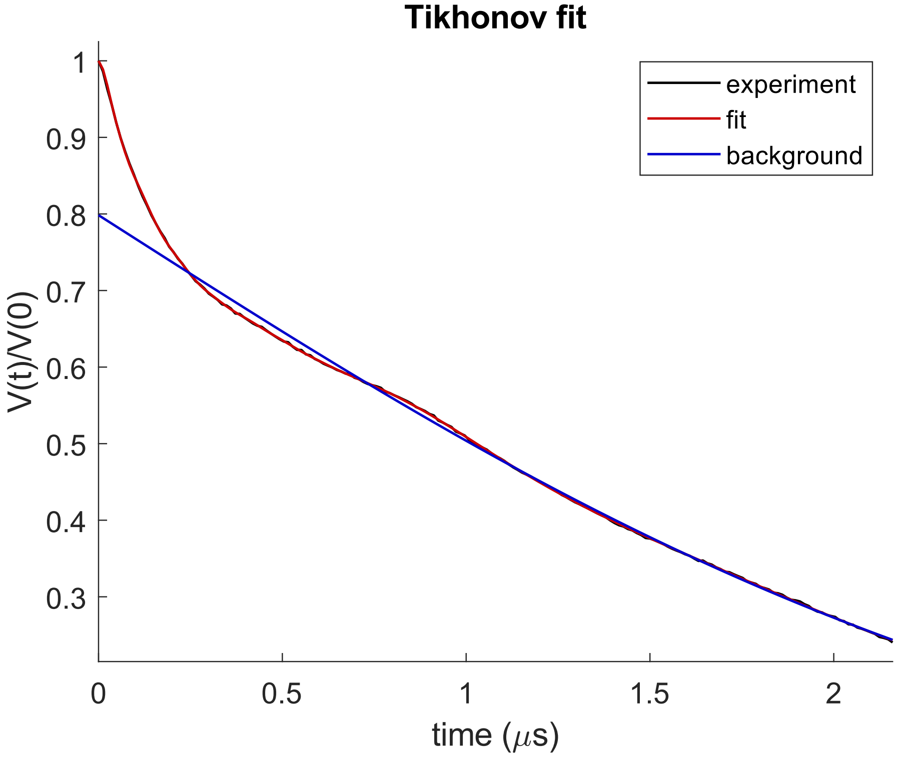

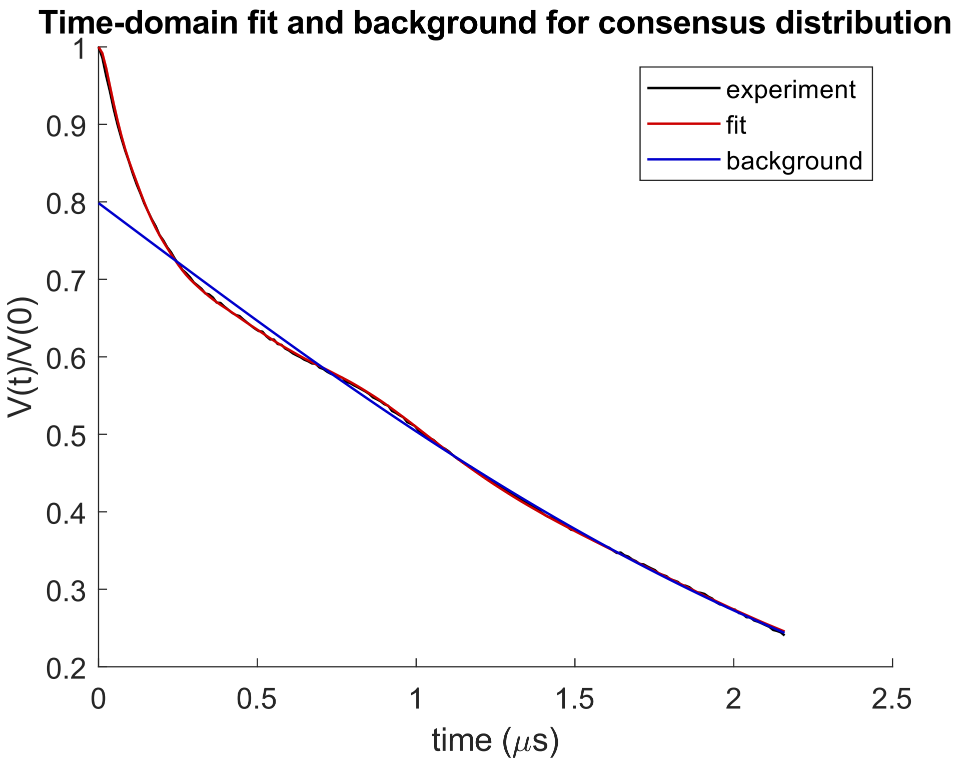
Time-domain fit and background for consensus distribution
experiment
fit
background
V(t)/V(0)
1
0.9
0.8
0.7
0.6
0.5
0.4
0.3
0.2
0
0.5
1
1.5
2
2.5
time (μs)

3. Experimental and processing parameters

RIDME processing was requested. Only DEERNet output.

Modulation depth: 0.201

Signal-to-noise ratio: 205.8 (w.r.t. modulation)

Noise estimates normalized to maximum signal

From imaginary part: 0.00000

From DEERNet fit: 0.00098

From Tikhonov fit: 0.00163

Zero time: 8 ns

Maximum time: 2160 ns

The last 3 % of the data was cut off

Time increment: 12 ns

Phase: 0.0 degree

Ensemble of 32 neural networks

Background separation by neural network

Background dimension: 3

Regularization parameter by best overlap with neural network solution

Regularization parameter used: 0.40

Reg. par. initial estimate by L-curve corner: 0.79

Overlap between DEERNet and regularization solutions: 0.928

Predicted overlap of consensus solution with ground truth: 0.80...0.97

Mean distance: 29.1 Å

Distance standard deviation: 6.8 Å

Full data set in Matlab format: C:\Users\ka44\Documents\OneDrive - University of St Andrews\StAndrews\Work\BEB\Projects\Albumin\HSA_EPR\HSA_Q\KAq205\205\230216_KAq205.9_RIDME_28_67_67000_comparative_DEER_analysis.mat

Distance distributions in text format: C:\Users\ka44\Documents\OneDrive - University of St Andrews\StAndrews\Work\BEB\Projects\Albumin\HSA_EPR\HSA_Q\KAq205\205\230216_KAq205.9_RIDME_28_67_67000_consensus_DEER_distribution.csv

Fit and background in text format: C:\Users\ka44\Documents\OneDrive - University of St Andrews\StAndrews\Work\BEB\Projects\Albumin\HSA_EPR\HSA_Q\KAq205\205\230216_KAq205.9_RIDME_28_67_67000_consensus_DEER_fit.csv

Metadata: C:\Users\ka44\Documents\OneDrive - University of St Andrews\StAndrews\Work\BEB\Projects\Albumin\HSA_EPR\HSA_Q\KAq205\205\230216_KAq205.9_RIDME_28_67_67000_comparative_DEER_meta_data.csv

DEER analysis report on dataset 230217_KAq205.15_RIDME_28_57_57000

DEERNet Spinach SVN Rev 5662 and DeerLab 0.9.1 Tikhonov regularization

ComparativeDEERAnalyzer version 2.0

see: S. G. Worswick et al., DOI: 10.1126/sciadv.aat5218, L. Fabregas Ibanez et al., DOI: 10.5194/mr-1-209-2020

02-Mar-2023 14:37:34

1. Distance distributions

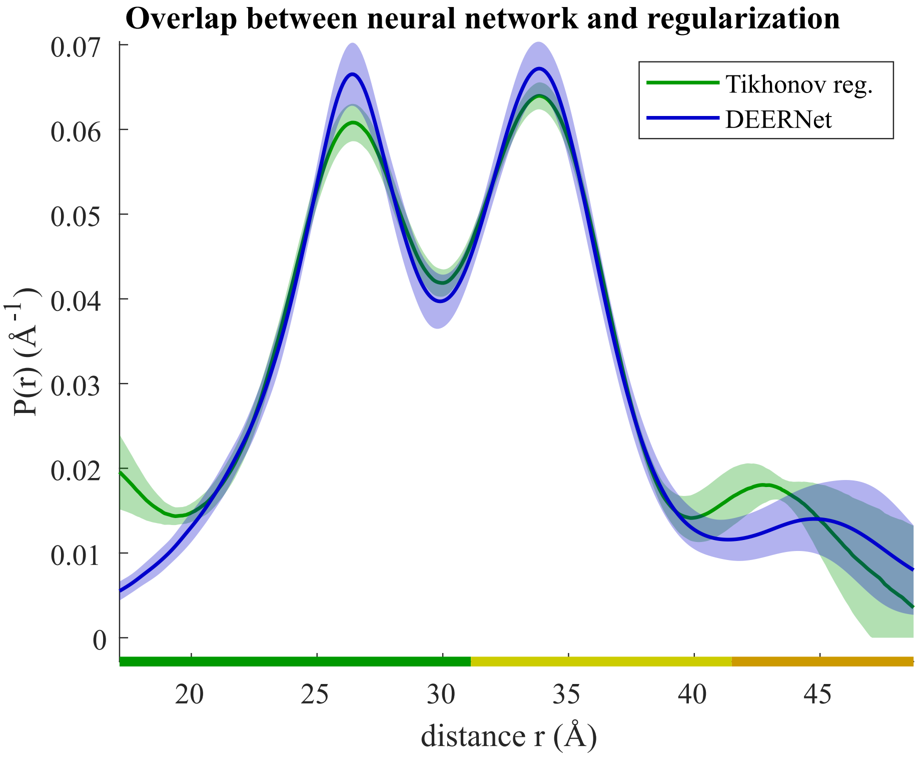

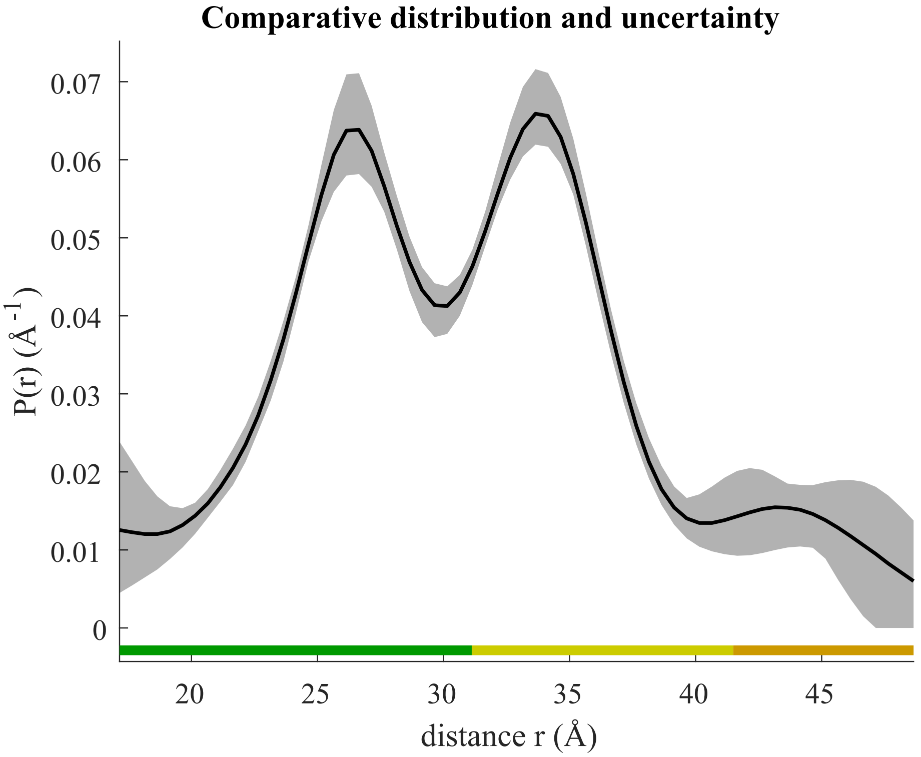

2. Fits of time-domain data

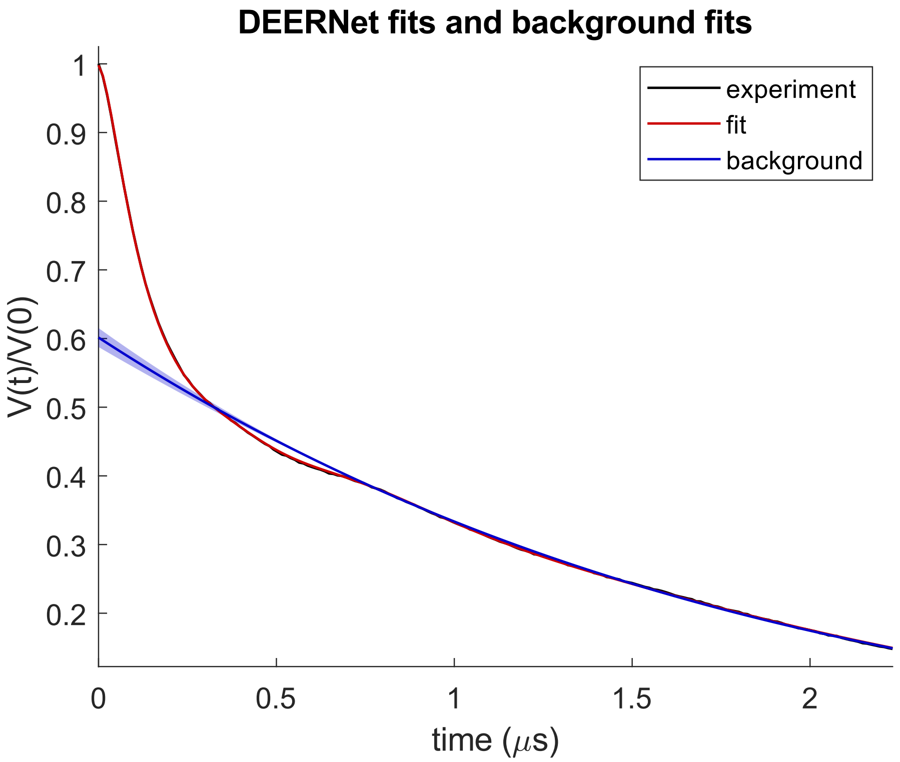

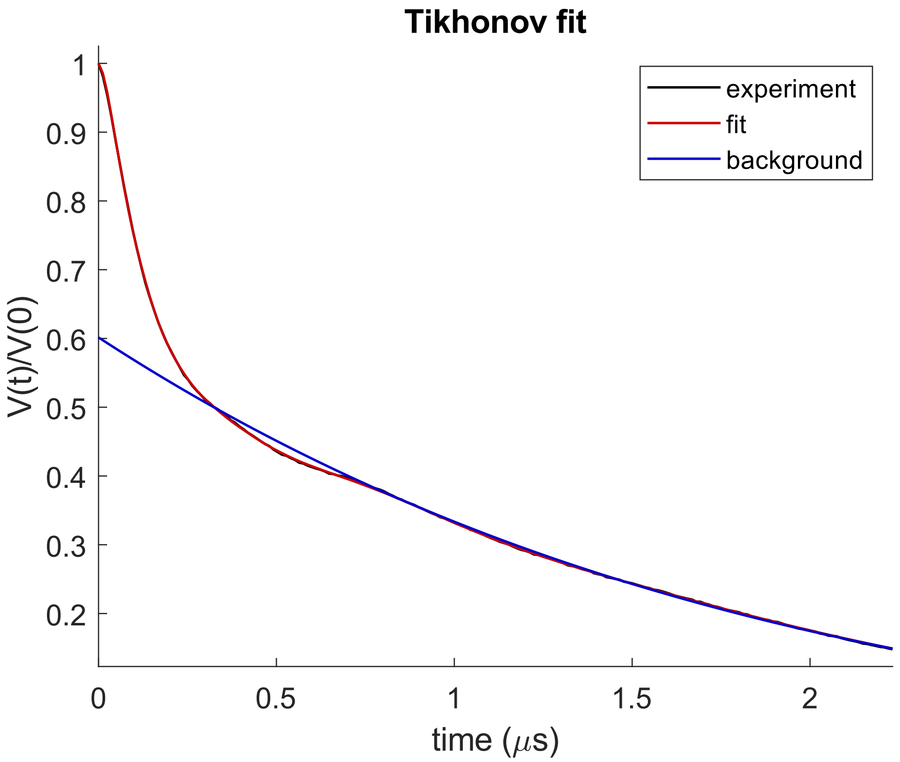

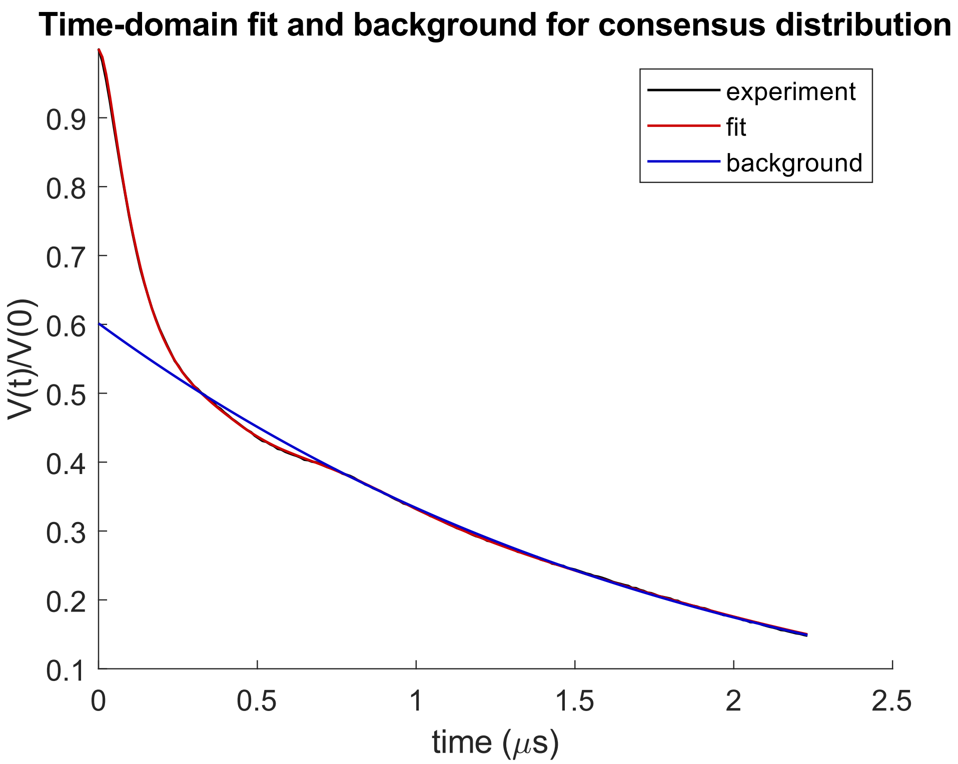
Time-domain fit and background for consensus distribution
experiment
fit
background
V(t)/V(0)
0.9
0.8
0.7
0.6
0.5
0.4
0.3
0.2
0.1
0
0.5
1
1.5
2
2.5
time (μs)

3. Experimental and processing parameters

RIDME processing was requested. Only DEERNet output.

Modulation depth: 0.397

Signal-to-noise ratio: 396.1 (w.r.t. modulation)

Noise estimates normalized to maximum signal

From imaginary part: 0.00000

From DEERNet fit: 0.00100

From Tikhonov fit: 0.00120

Zero time: 9 ns

Maximum time: 2232 ns

Time increment: 12 ns

Phase: 0.0 degree

Ensemble of 32 neural networks

Background separation by neural network

Background dimension: 3

Regularization parameter by best overlap with neural network solution

Regularization parameter used: 2.83

Reg. par. initial estimate by L-curve corner: 1.00

Overlap between DEERNet and regularization solutions: 0.958

Predicted overlap of consensus solution with ground truth: 0.82...0.99

Mean distance: 30.4 Å

Distance standard deviation: 7.8 Å

Full data set in Matlab format: C:\Users\ka44\Documents\OneDrive - University of St Andrews\StAndrews\Work\BEB\Projects\Albumin\HSA_EPR\HSA_Q\KAq205\205\230217_KAq205.15_RIDME_28_57_57000_comparative_DEER_analysis.mat

Distance distributions in text format: C:\Users\ka44\Documents\OneDrive - University of St Andrews\StAndrews\Work\BEB\Projects\Albumin\HSA_EPR\HSA_Q\KAq205\205\230217_KAq205.15_RIDME_28_57_57000_consensus_DEER_distribution.csv

Fit and background in text format: C:\Users\ka44\Documents\OneDrive - University of St Andrews\StAndrews\Work\BEB\Projects\Albumin\HSA_EPR\HSA_Q\KAq205\205\230217_KAq205.15_RIDME_28_57_57000_consensus_DEER_fit.csv

Metadata: C:\Users\ka44\Documents\OneDrive - University of St Andrews\StAndrews\Work\BEB\Projects\Albumin\HSA_EPR\HSA_Q\KAq205\205\230217_KAq205.15_RIDME_28_57_57000_comparative_DEER_meta_data.csv

DEER analysis report on dataset 230216_KAq205.12_RIDME_26_55_55000

DEERNet Spinach SVN Rev 5662 and DeerLab 0.9.1 Tikhonov regularization

ComparativeDEERAnalyzer version 2.0

see: S. G. Worswick et al., DOI: 10.1126/sciadv.aat5218, L. Fabregas Ibanez et al., DOI: 10.5194/mr-1-209-2020

02-Mar-2023 14:14:48

1. Distance distributions

Overlap between neural network and regularization

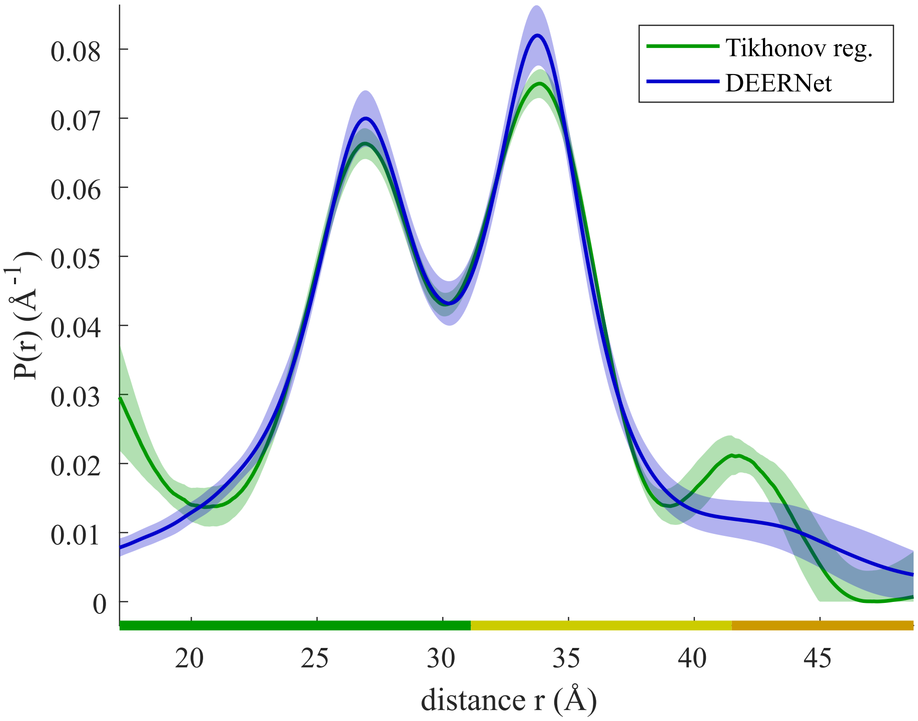

Comparative distribution and uncertainty

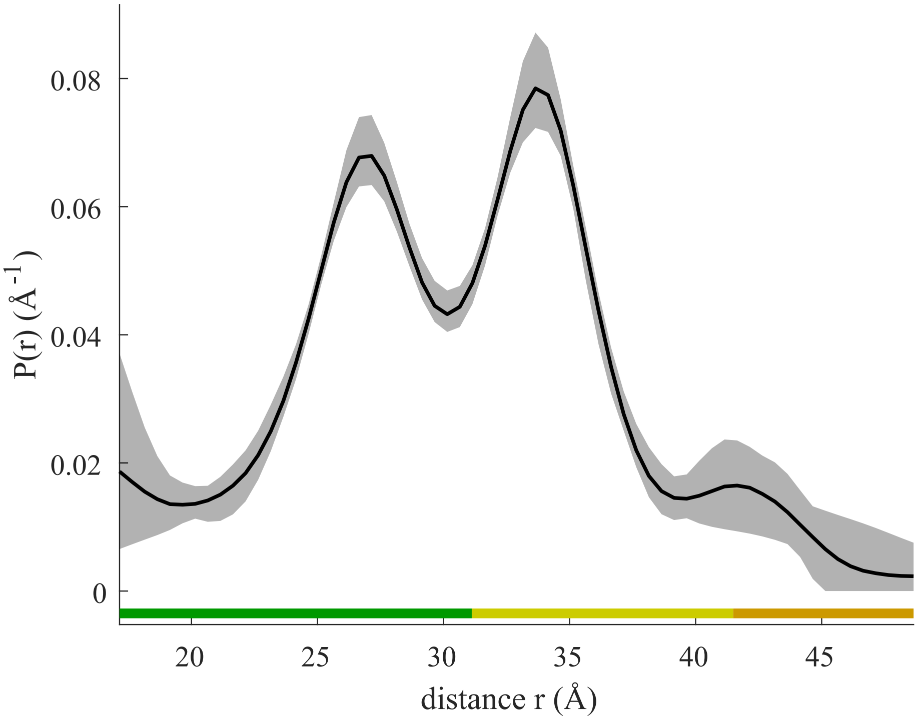

2. Fits of time-domain data

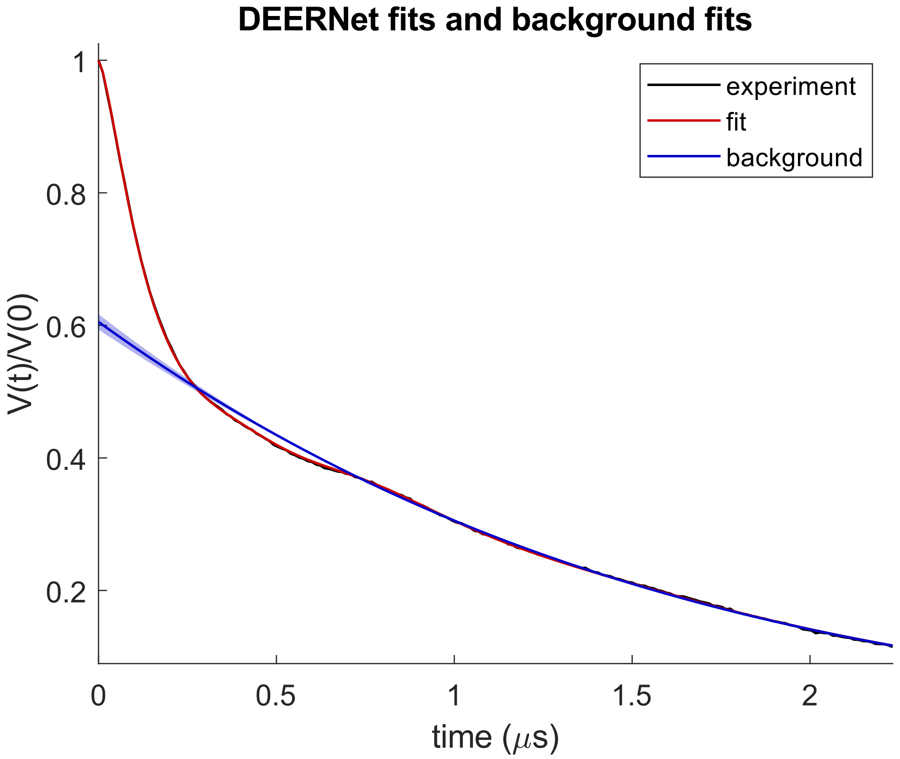

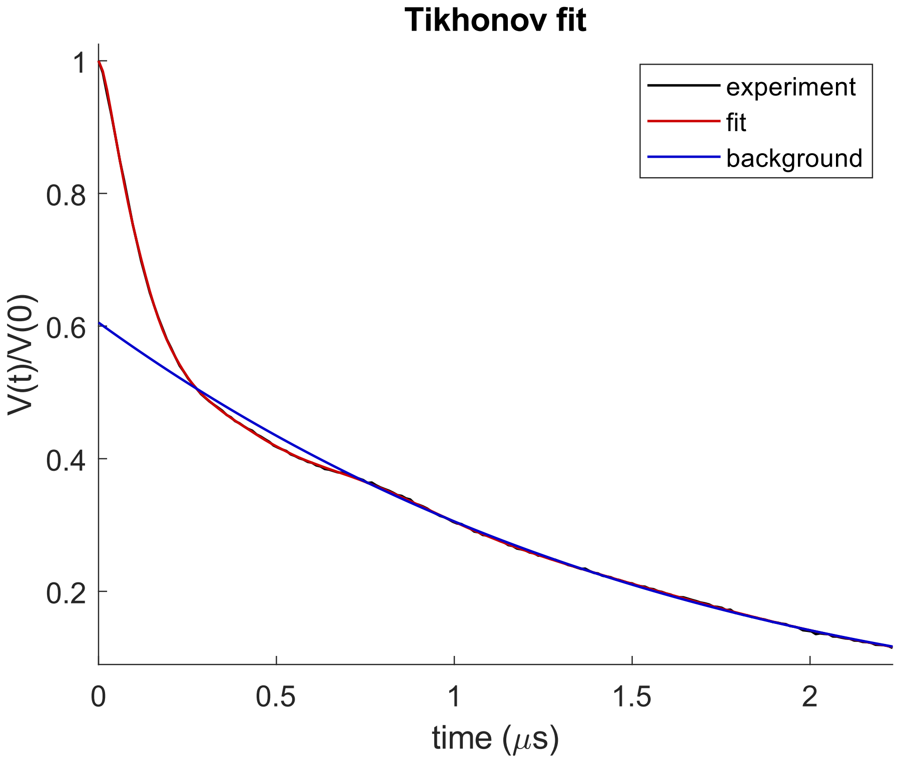

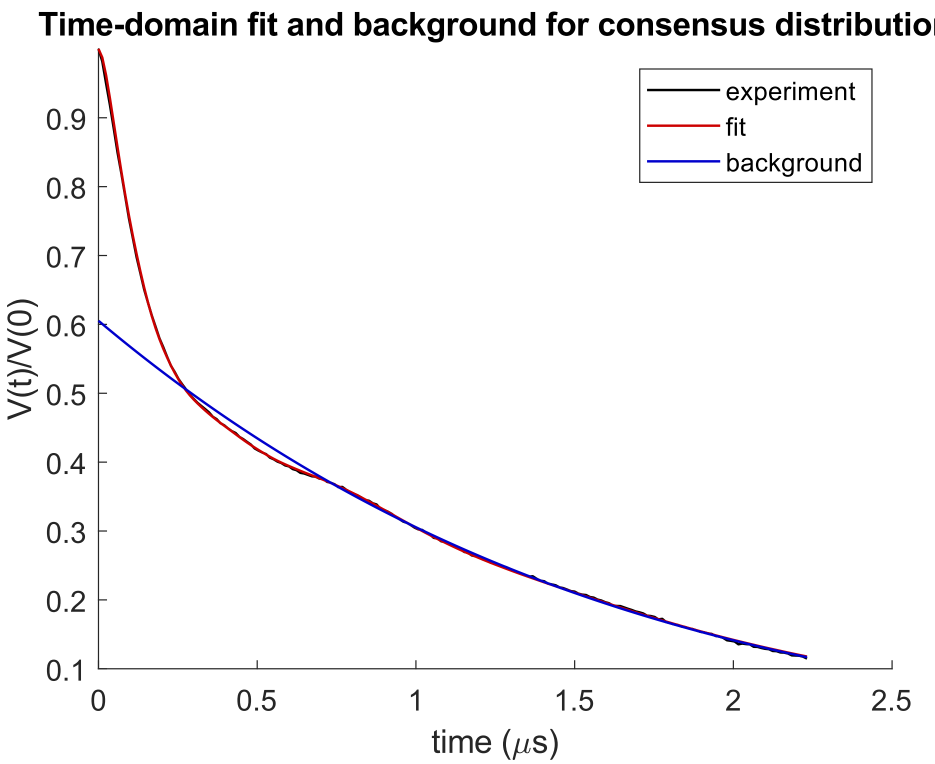
Time-domain fit and background for consensus distribution
experiment
fit
background
V(t)/V(0)
0.1
0.2
0.3
0.4
0.5
0.6
0.7
0.8
0.9
0
0.5
1
1.5
2
2.5
time (μs)

3. Experimental and processing parameters

RIDME processing was requested. Only DEERNet output.

Modulation depth: 0.395

Signal-to-noise ratio: 306.0 (w.r.t. modulation)

Noise estimates normalized to maximum signal

From imaginary part: 0.00000

From DEERNet fit: 0.00129

From Tikhonov fit: 0.00152

Zero time: 9 ns

Maximum time: 2232 ns

Time increment: 12 ns

Phase: 0.0 degree

Ensemble of 32 neural networks

Background separation by neural network

Background dimension: 3

Regularization parameter by best overlap with neural network solution

Regularization parameter used: 1.78

Reg. par. initial estimate by L-curve corner: 1.26

Overlap between DEERNet and regularization solutions: 0.940

Predicted overlap of consensus solution with ground truth: 0.81...0.98

Mean distance: 30.4 Å

Distance standard deviation: 6.7 Å

Full data set in Matlab format: C:\Users\ka44\Documents\OneDrive - University of St Andrews\StAndrews\Work\BEB\Projects\Albumin\HSA_EPR\HSA_Q\KAq205\205\230216_KAq205.12_RIDME_26_55_55000_comparative_DEER_analysis.mat

Distance distributions in text format: C:\Users\ka44\Documents\OneDrive - University of St Andrews\StAndrews\Work\BEB\Projects\Albumin\HSA_EPR\HSA_Q\KAq205\205\230216_KAq205.12_RIDME_26_55_55000_consensus_DEER_distribution.csv

Fit and background in text format: C:\Users\ka44\Documents\OneDrive - University of St Andrews\StAndrews\Work\BEB\Projects\Albumin\HSA_EPR\HSA_Q\KAq205\205\230216_KAq205.12_RIDME_26_55_55000_consensus_DEER_fit.csv

Metadata: C:\Users\ka44\Documents\OneDrive - University of St Andrews\StAndrews\Work\BEB\Projects\Albumin\HSA_EPR\HSA_Q\KAq205\205\230216_KAq205.12_RIDME_26_55_55000_comparative_DEER_meta_data.csv

DEER analysis report on dataset 230618_BEBQ82.6_RIDME

DEERNet Spinach SVN Rev 5662 and DeerLab 0.9.1 Tikhonov regularization

ComparativeDEERAnalyzer version 2.0

see: S. G. Worswick et al., DOI: 10.1126/sciadv.aat5218, L. Fabregas Ibanez et al., DOI: 10.5194/mr-1-209-2020

13-Sep-2023 14:58:00

1. Distance distributions

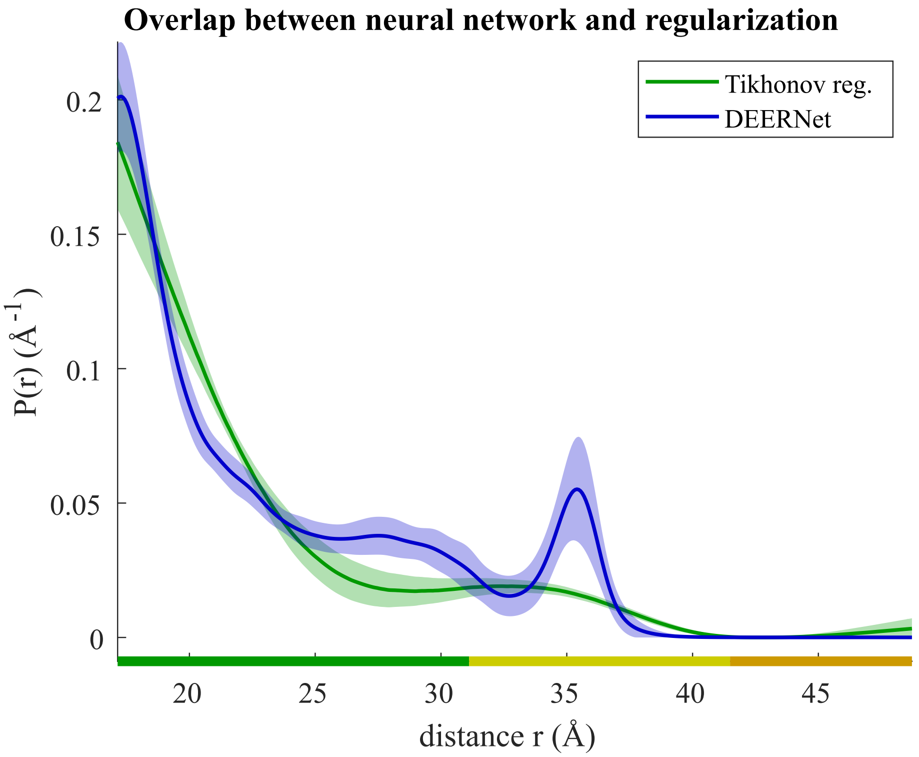

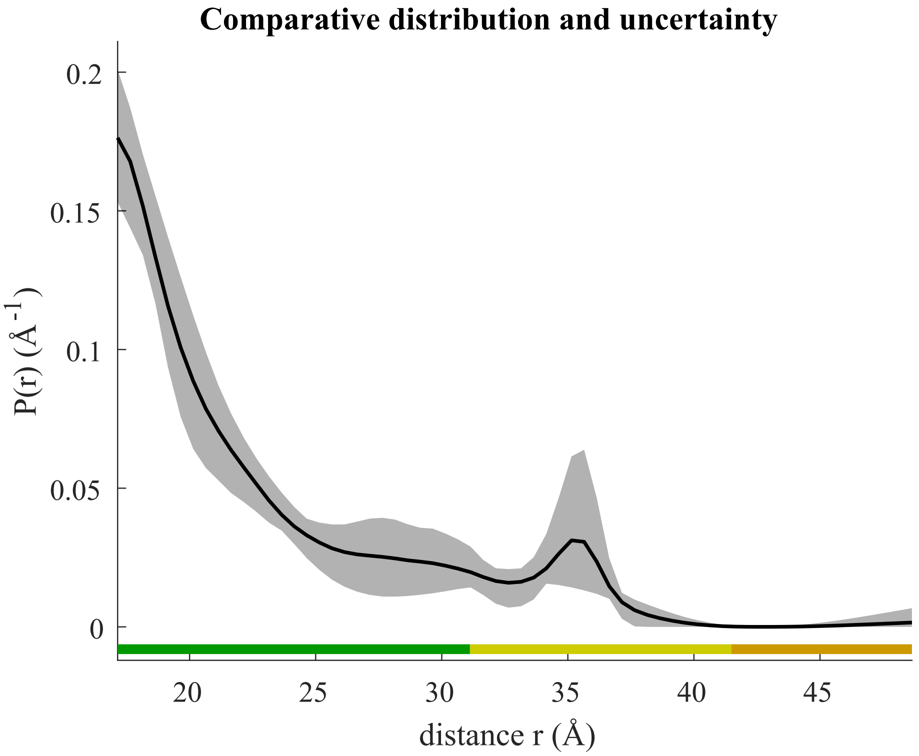

2. Fits of time-domain data

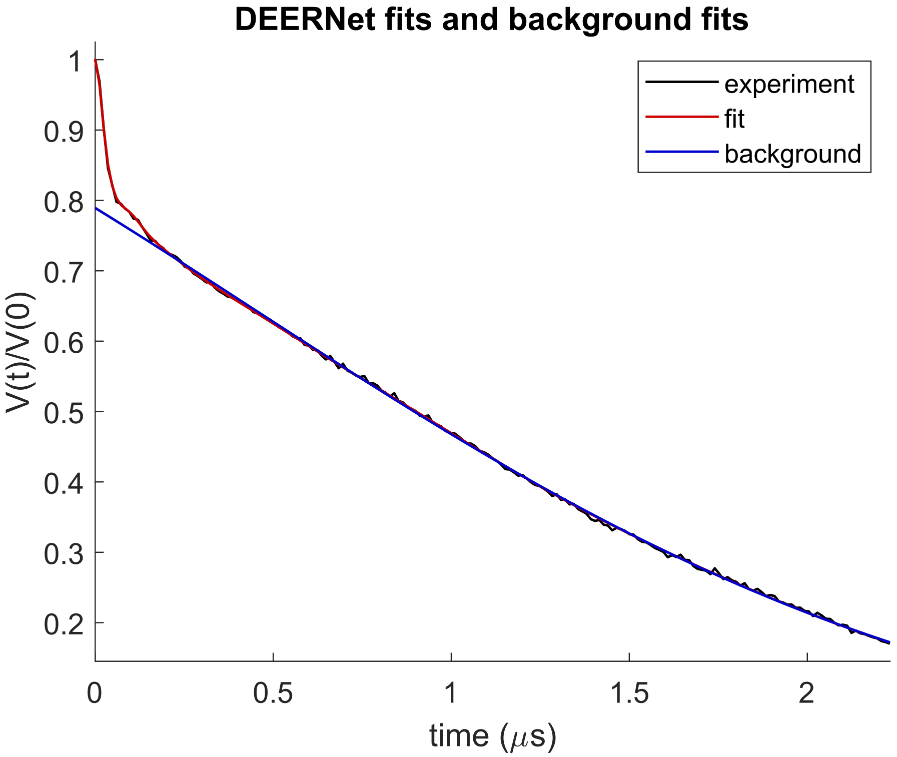

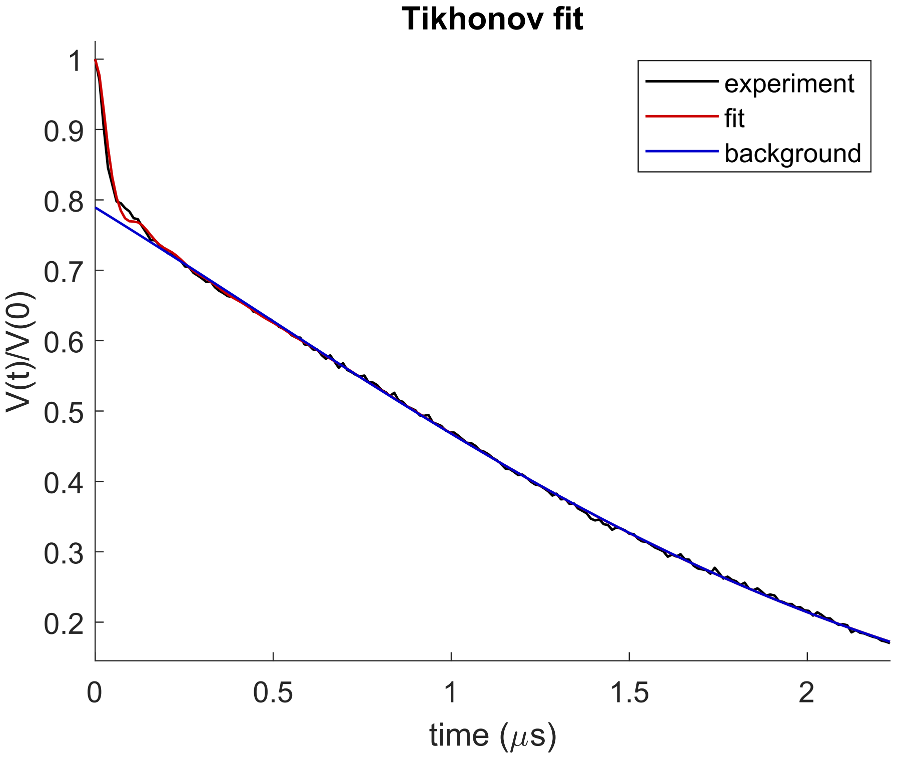

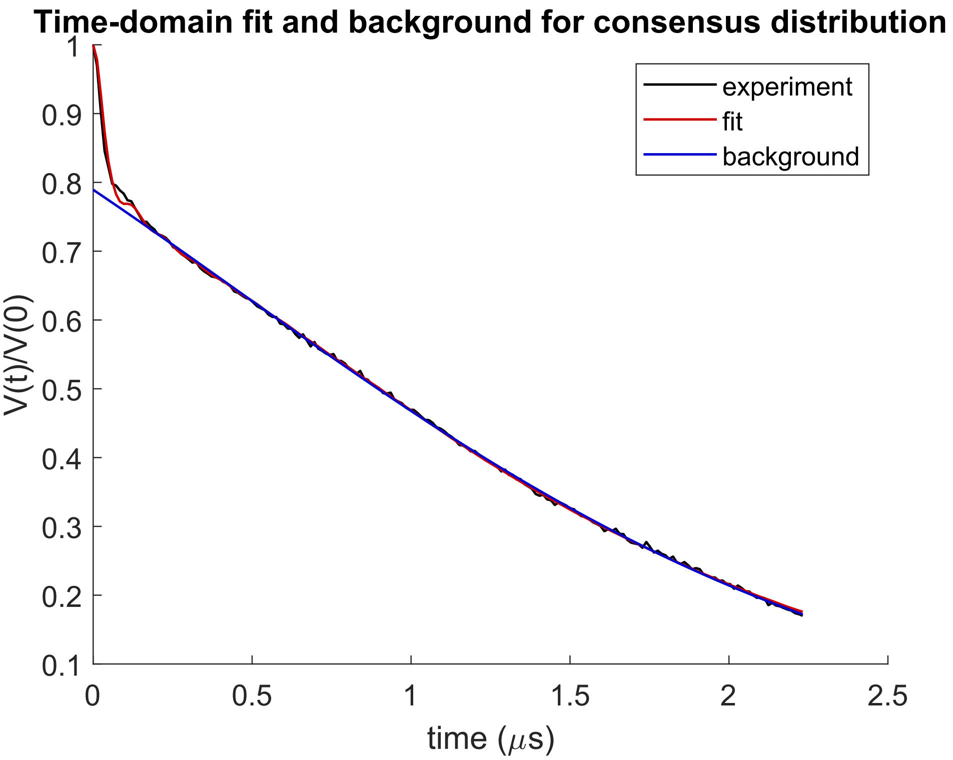
Time-domain fit and background for consensus distribution
experiment
fit
background
V(t)/V(0)
1
0.9
0.8
0.7
0.6
0.5
0.4
0.3
0.2
0.1
0
0.5
1
1.5
2
2.5
time (μs)

3. Experimental and processing parameters

RIDME processing was requested. Only DEERNet output.

Modulation depth: 0.211

Signal-to-noise ratio: 78.7 (w.r.t. modulation)

Noise estimates normalized to maximum signal

From imaginary part: 0.00346

From DEERNet fit: 0.00269

From Tikhonov fit: 0.00496

Zero time: 14 ns

Maximum time: 2232 ns

Time increment: 12 ns

Phase: 1.7 degree

Ensemble of 32 neural networks

Background separation by neural network

Background dimension: 3

Regularization parameter by best overlap with neural network solution

Regularization parameter used: 15.96

Reg. par. initial estimate by L-curve corner: 2.00

Overlap between DEERNet and regularization solutions: 0.861

Predicted overlap of consensus solution with ground truth: 0.73...0.90

Mean distance: 23.1 Å

Single Gaussian provided different mean distance. Distribution may be incomplete.

Distance standard deviation: 6.0 Å

Full data set in Matlab format: C:\Users\Katrin\OneDrive - University of St Andrews\StAndrews\Work\BEB\Projects\Albumin\HSA_EPR\HSA_Q\H288A_2eqCu\230618_BEBQ82.6_RIDME_comparative_DEER_analysis.mat

Distance distributions in text format: C:\Users\Katrin\OneDrive - University of St Andrews\StAndrews\Work\BEB\Projects\Albumin\HSA_EPR\HSA_Q\H288A_2eqCu\230618_BEBQ82.6_RIDME_consensus_DEER_distribution.csv

Fit and background in text format: C:\Users\Katrin\OneDrive - University of St Andrews\StAndrews\Work\BEB\Projects\Albumin\HSA_EPR\HSA_Q\H288A_2eqCu\230618_BEBQ82.6_RIDME_consensus_DEER_fit.csv

Metadata: C:\Users\Katrin\OneDrive - University of St Andrews\StAndrews\Work\BEB\Projects\Albumin\HSA_EPR\HSA_Q\H288A_2eqCu\230618_BEBQ82.6_RIDME_comparative_DEER_meta_data.csv
